# Bacterial consortia enhance nutrient uptakes and molecular response in tomato seedlings under alkaline soil stress: a comparative study

**DOI:** 10.3389/fmicb.2026.1738650

**Published:** 2026-02-26

**Authors:** Keerthana Rangasamy, Arabi Mohammed Saleh

**Affiliations:** 1 School of Bioscience and Technology, Vellore Institute of Technology, Vellore, India; 2 VIT School of Agricultural Innovations and Advanced Learning, Vellore Institute of Technology, Vellore, India

**Keywords:** alkaline stress, bacterial consortia, enzyme activity, gene expression, seed germination

## Abstract

Nutrient deficiencies in alkaline soils (pH 7.9–8.5) frequently limit plant growth due to insufficient nutrient availability and uptake. This study investigated the effects of two bacterial strains, VITK-1 (*Pseudomonas* sp.) and VITK-3 (*Burkholderia* sp.), on nutrient absorption, growth, and gene expression in tomato (*Solanum lycopersicum*) seedlings grown in alkaline soil. Bacterial treatments were applied individually and as a consortium, and their ability to promote plant growth and nutrient solubility was evaluated. *In vitro* studies demonstrated the strains’ ability to solubilize essential nutrients, generate extracellular enzymes, and exhibit a variety of Plant Growth-Promoting Rhizobacteria (PGPR) characteristics, with strong antagonistic activity against *Fusarium oxysporum f.*sp. *lycopersici* and *Ralstonia solanacearum* (35.7%–76.5%). *In vivo* investigations revealed notable improvements in germination (73.3%), root and shoot development, and overall seedling vigor when compared to untreated controls. The bacterial consortium significantly improved protein (54.5%) and proline (69.5%) levels, antioxidant activity (50.7%), phenolic (60.9%), and flavonoid content (52.5%), and decreased carbohydrate accumulation. Furthermore, treated plants exhibited activation of nutrient-regulating genes (*NRT2*, *PR-1*, and *AMT-1*) associated with better root metabolism (improved 1.58–1.70 mg) and resilience to stress (*GR-1* and *DREB3*). These results show the potential of PGPR inoculants, particularly consortia, as a promising strategy for improving nutrient uptake, biochemical characteristics, and stress tolerance in crops grown in alkaline soils.

## Graphical abstract

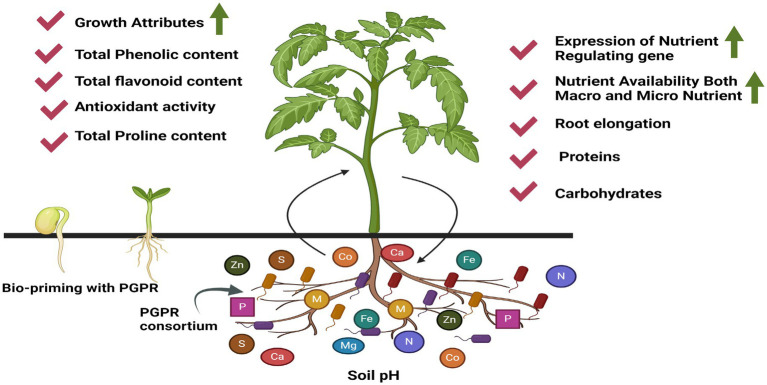

## 1 Introduction

Tomato (*Solanum lycopersicum*) is a highly valued and widely consumed food crop worldwide. It belongs to the Solanaceae family, which includes tomato and potato (Paungfoo-Lonhienne et al., 2016; Salehi et al., 2019). Tomato price volatility has gained global attention, with prices rising by 64.46% in June 2023 (FAO, 2021; Advance and Welfare, 2023). India produces approximately 215.49 lakh tonnes of tomatoes annually, with Tamil Nadu contributing 6%–7%, while global production stands at nearly 189 million tonnes (Vijayakumar et al., 2021). Renowned for its nutritional, medicinal, and physiological benefits, the tomato is rich in vitamins, lycopene, beta-carotene, amino acids, and essential phytosterols, such as campesterol and stigmasterol (Abdullahi et al., 2016; Gashash et al., 2022). Its widespread popularity has led to extensive cultivation and large-scale production worldwide, with numerous studies highlighting its economic and nutritional significance (Arah et al., 2016; Park et al., 2020).

Soil pH is a critical determinant of nutrient availability in agricultural systems, directly influencing plant growth and development (Läuchli and Grattan, 2012). Alkaline soil conditions pose significant challenges to crop production, as they restrict the availability of essential nutrients like phosphorus, iron, and zinc (Msimbira and Smith, 2020). Prolonged irrigation in alkaline conditions exacerbates these issues by increasing the bicarbonate content (Sunaratiya and Kapgate, 2021), contributing to soil salinity, impairing plant development, and degrading soil health. High soil pH disrupts root cell structure, triggers metabolic imbalances, and reduces nutrient absorption, leading to impaired microbial activity, organic matter decomposition, nutrient cycling, and overall crop yield (Li et al., 2020; Zhou et al., 2024). Additionally, alkaline soil conditions alter plants’ natural defense mechanisms, induce osmotic imbalance, and disrupt physiological metabolism (Rasheed et al., 2022). Monitoring soil pH and electrical conductivity before cultivation is essential for assessing nutrient and mineral availability, thereby facilitating informed agricultural decisions (Shrivastava and Kumar, 2015).

To mitigate the adverse effects of alkaline soils, sustainable, viable farming techniques such as the application of plant growth-promoting rhizobacteria (PGPR) have gained attention. By improving nutrient uptake, enhancing stress tolerance, and interacting with other soil organisms under abiotic stress conditions like drought, salinity, and significant temperatures, PGPR are beneficial soil bacteria and promote plant growth (Singh, 2018). While numerous studies have extensively demonstrated the general benefits of PGPR in promoting plant growth, their application in alkaline soils remains underexplored due to limited resources, which is addressed in the present study. Compared to single bacterial inoculants, PGPR-based bacterial consortia have a particularly synergistic effect on plant growth due to their enhanced functional capabilities, including increased crop yield, nutrient solubilization, biocontrol activity, maintenance of soil health under high pH conditions, and stabilization of long-term rhizobacterial interactions. However, despite these advantages, limited research has been done on the combined use of PGPR consortia in alkaline soil conditions (Singh et al., 2023b). Previous studies indicate that PGPR strains suppress plant pathogens through the activation of induced systemic resistance, a mechanism that may be particularly effective in alkaline soil conditions, where pathogen expression may differ during experimental conditions (Egamberdieva et al., 2019; Wang et al., 2021). In addition, the synthesis of extracellular enzymes like cellulase, proteases and lipase directly inhibits phytopathogens by degrading fungal cell walls and indirectly improves nutrient accumulation via enhanced root metabolic activity, thereby promoting plant growth and development rather than pathogen suppression, mediated by hydrolytic enzymatic activity (Mazuecos-Aguilera et al., 2025). Consequently, there is growing interest in investigating the potential of PGPR to improve soil fertility and enhance the physiological and biochemical responses of tomato seedlings in high-pH environments (Jeyanthi and Kanimozhi, 2018). Although most studies have extensively reported the effects of individual PGPR strains and non-alkaline stress conditions, research on PGPR-mediated consortia under alkaline soil conditions remains limited, particularly in terms of nutrient uptake and microbial stability at high soil pH. It is currently unknown how PGPR’s combined efficiency improves microbial stability, nutrient accumulation, and antioxidant activity through the enzyme production and molecular response to stress tolerance. This will improve the critical gap regarding the combined microbial strategies in alkaline soil.

Given their nutritional significance, tomatoes are an ideal crop for enhancing nutrient content through treatments such as plant growth promoting rhizobacteria (PGPR), particularly in alkaline soil conditions. PGPR, often referred to as the root microbiome, has demonstrated its ability to promote vegetative growth, accelerate the absorption of water and electrolytes, and anchor crucial minerals and amino acids from the soil, thereby improving plant growth (Khoso et al., 2024). In various climatic conditions, PGPR applications have proven to be a promising strategy in increasing plant growth and early nutrient uptake by metabolizing both organic and inorganic compounds derived from environmental sources. Beneficial strains of PGPR can enhance plant growth, detoxify heavy metals, produce siderophores and indole-3-acetic acid (IAA), and increase the shoot and root biomass, thus acting as phytostimulants (Sayyed, 2019). Among many PGPR strains, *Burkholderia* and *Paraburkholderia* are notable for promoting plant growth by producing auxin, fixing nitrogen, solubilizing phosphorus, and efficiently protecting host plants from abiotic stress (Pal et al., 2022). Newly isolated *Pseudomonas* species from agroecological environments have demonstrated their ability to solubilize phosphate, ammonia, and siderophores, colonize tomato roots, improve nutrient uptake, and suppress pathogens (Qessaoui et al., 2019). Similarly, *Burkholderia cepacia* produces secondary metabolites and accelerates rhizosphere colonization, aiding stress tolerance (Jung et al., 2018). Considering the widespread cultivation of tomatoes, optimizing plant nutrients with fertilizer applications is crucial for commercial agricultural purposes (Kalozoumis et al., 2021; Gashash et al., 2022). Through increased resilience and adaptive responses, PGPRs help plants survive temperature-induced stress even though they do not control external temperatures (Ferreira et al., 2019). While extensive research has been conducted on microbial responses to saline-alkaline stress, studies on the combined effects of *Pseudomonas* sp. VITK-1 and *Burkholderia* sp. VITK-3 under alkaline soil conditions remain limited. This study explores how these bacterial consortia influence plant physiological parameters, as well as micro- and macronutrient uptake in tomato roots under stress conditions. Additionally, we examined the regulation of nutrient and stress-related genes in tomato roots and investigated biochemical defense mechanisms that enable plants to withstand abiotic stress. This study is to demonstrate significant changes in tomato seedling responses when seeds were treated with bacterial consortia and individual strains, particularly concerning physiological and biochemical parameters associated with soil-root microbial interactions.

In this study, we have identified the potential PGPR candidates and evaluated their consortia effects on tomato seedling growth under alkaline soil conditions. Specifically, we assessed nutrient uptake, stress tolerance responses, and biochemical changes in tomato seedlings. Additionally, we examined germination rates and analyzed gene expression related to nutrient absorption, enduring stress, and stimulation for the growth of tomato roots. The results provide novel insights concerning how PGPR might enhance sustainable agricultural practices, emphasizing their role in addressing nutrient limitation and improving crop resilience under stressful environmental conditions.

## 2 Materials and methods

### 2.1 Sample collection

A total of two different soil samples were collected from the rhizosphere of tomato plants within a depth of 3 cm. Each sample weighed 10 g and was collected from tomato plants grown in two different experimental fields within the agricultural area at Vellore Institute of Technology, Vellore, Tamil Nadu, India. The samples were packaged in polythene bags and delivered to the lab for *in vitro* testing.

### 2.2 Culture conditions and maintenance

**Figure 1:**
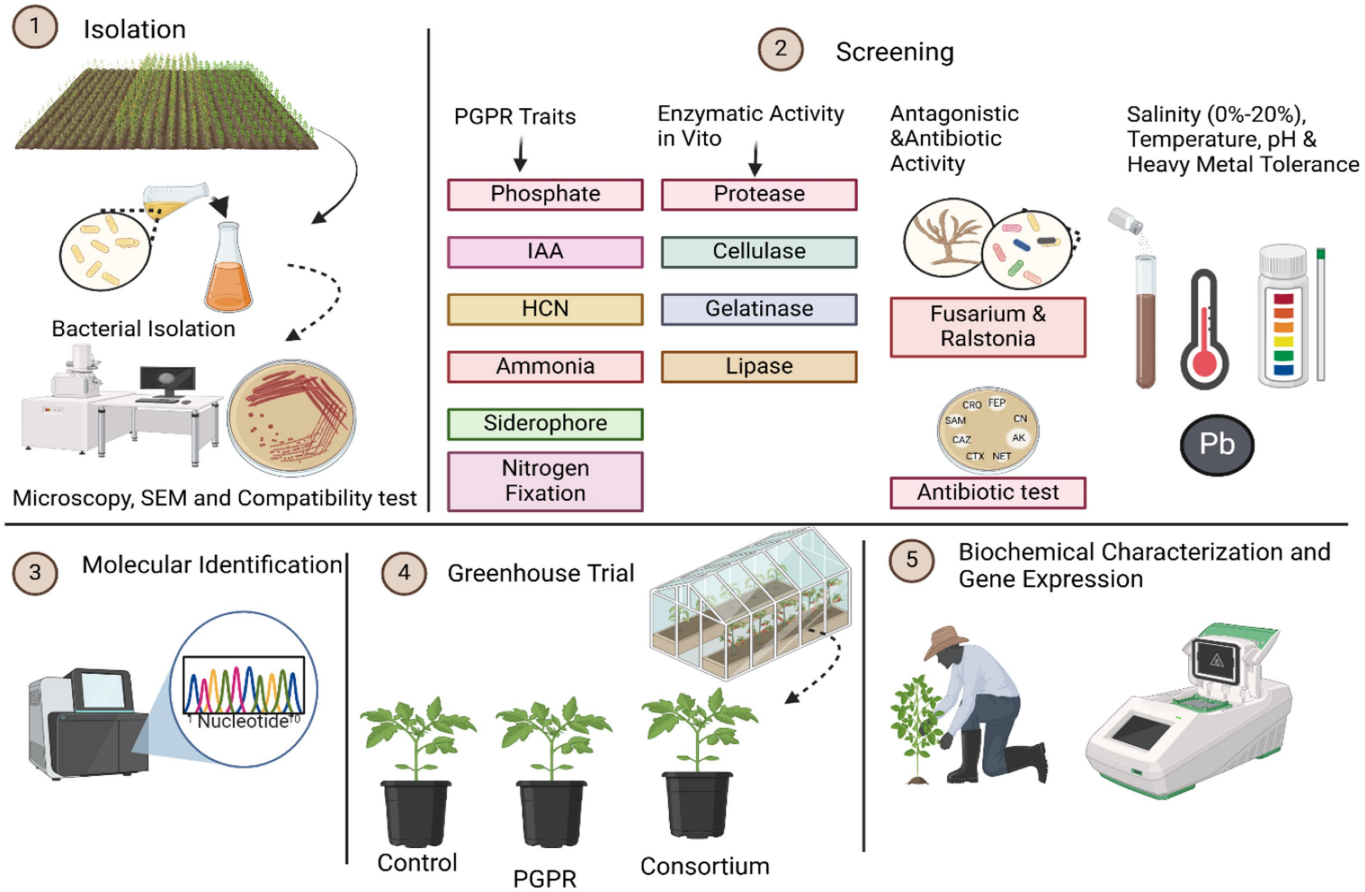
Schematic overview of the research process: the figure illustrates the sequential steps involved in the isolation and characterization of selected bacterial strains. These isolates were further screened for *in vitro* enzymatic and antagonistic activity, as well as for tolerance to salinity, temperature, pH, and heavy metals. The identified strains were tested before greenhouse trials to assess plant growth. The treatments were designated as control, individual, and consortium treatments. In addition, plant physiological and biochemical characterization, as well as gene expression, were performed to evaluate significant growth enhancement, respectively. 1. Isolation of bacteria, 2. Screening of PGPR test, 3. Molecular identification of isolates, 4. Greenhouse trail, 5. Biochemical characterization and gene expression.

These soil samples were serially diluted (up to 10^−7^ dilutions) using sterile distilled water and were subsequently spread plated on fresh Nutrient agar plates for bacterial isolation. The plates were incubated at 37 °C for 24 h. The initial soil samples were preserved in a refrigerator at below 10 °C. The isolates were sub-cultured at alternate intervals of 15 days using the nutrient broth and were maintained at 50% glycerol stock (−20 °C) for future use (Jung et al., 2018; Andy et al., 2020). Morphological observations of the strains were conducted using scanning electron microscopy and phase-contrast microscopy. The research process’s schematic overview is shown in Figure 1.

### 2.3 Screening of PGPR traits

Phosphate solubilization ability of the isolates was evaluated using National Botanical Research Institute’s Phosphate (NBRIP) medium. Each of the isolates was inoculated into well-made up centers of fresh NBRIP plates and were incubated at 30 °C for 48 h. After incubation, the plates were examined for any clear zone surrounding the colonies, indicating the isolates may solubilize phosphate (Shektar, 1999; Qingwei et al., 2023). To assess the ability to synthesize Indole-3-Acetic Acid (IAA), Tryptophan was added to the cultured nutrient broth and incubated at 37° C under shaking conditions (150 rpm). After incubation, the bacterial cultures were centrifuged at 5000 rpm for 10 min to collect the cell-free supernatant. Salkowski reagent (57 milliliters of 60% perchloric acid, 4 milliliters of double distilled water, and 2 milliliters of 0.5 M FeCl_3_) was mixed with 1 milliliter of the aforementioned cell-free supernatant (Madhurama et al., 2014), and the absorbance was measured at 530 nm using a UV–Vis spectrophotometer (Shimadzu) (Al-Rashid, 2013).

Chrome azurol sulfonate (CAS) was used to detect siderophore formation, following the procedure detailed by Schwyn and Neilands (1987), with slight modifications. Cultures exhibiting a yellowish-orange ring outside after 48 h of incubation indicate the positive production of siderophore. Ammonia production was tested by incubating the isolates in a peptone and sodium chloride medium. The appearance of a yellowish-orange color upon adding Nessler’s reagent indicated ammonia generation using the Cappuccino and Sherman (1992) method (Mukherjee et al., 2017; Gohil et al., 2022).

Hydrogen cyanide (HCN) production was assessed by inoculating the culture into a glycine nutrient-enriched medium, with minor modifications from the method described by Bakker and Schippers (1987) with minor modifications (Kumar et al., 2014). The medium was pre-treated with a sodium carbonate solution and picric acid to aid colour development. The filter paper was then sealed with parafilm to provide consistent environmental conditions. After 4 days of incubation at room temperature, the plates were observed for any orange pigmentation, indicating the effectiveness of ammonia production. Lastly, the nitrogen-fixing properties of the isolates were evaluated using Jenen’s medium (Reang et al., 2022). The bacterial cultures were streaked onto plates with pre-prepared medium and was incubated for 2 days at 37 °C. The plates were observed for any growth following incubation.

### 2.4 16s rDNA sequencing and PGPR characterization

The bacterial isolates were subjected to DNA extraction, sequencing, and analysis using the Basic Local Alignment Search Tool (BLAST). Whole genomic DNA was extracted from pure cultures using the Xploregen Universal Extraction Kit (Xploregen Discoveries, India). A 1.5 kbp segment of the 16s rDNA gene was amplified using high-fidelity PCR polymerase and the primers 16s Forward- GGATGAGCCCGCGGCCTA (72.22% GC content) and 16 s Reverse- CGGTGTGTACAAGGCCCGG (68.42% GC content). After thermal cycling was completed using a BIO-RAD T100 thermal cycler set to anneal at 58 °C, the PCR product was sequenced in both directions.

Primer concentrations of 10 pM were used to amplify up to 126 ng of isolated DNA. High-Fidelity DNA polymerase, 5 nM DNTPs, 3.2 mM MgCl2, and PCR Enzyme Buffer made up the TAQ Master mix. Thirty cycles of denaturation for 1 min at 94 °C, annealing for 1 min at 50 °C, and extension for 2 mins at 72 °C comprised the PCR cycling process. The initial denaturation step lasted 3 mins at 94 °C. For 7 mins, the last extension stage was conducted at 72 °C. After the PCR product was sequenced and subjected to similarity analysis using BLAST (Basic Local Alignment Search Tool) and NCBI (National Center for Biotechnology Information) the sequence was added to the Gene Bank database along with accession numbers (Stephen and Saleh, 2023). Phylogenetic analysis for the strains (VITK-1 and VITK-3) and 16s rRNA fragment amplification were carried out by Bio Kart India, Pvt. Ltd., Bengaluru.

### 2.5 Screening of hydrolytic enzymatic activity

The bacterial strains were assessed for extracellular enzymatic activity to enhance plant growth and development. Hydrolytic enzyme assays were conducted as the primary screening to evaluate the biocontrol activity of the PGPR strains. These tests provide functional validation of antagonistic activity, which refers to PGPR characteristics. For Protease production, isolates were cultured in the nutrient broth, and then the cells were centrifuged at 1000 rpm for 10 min at 4 °C. Twenty microliters of the cell-free supernatants were poured into a medium composed of skimmed agar (Alnahdi, 2012; Sritongon et al., 2023) and incubated for 48 h to observe for any clear zone.

Cellulose production was identified using the method described by Ariffin et al. (2006) and Samira et al. (2010). Cultures were incubated for 48 h, followed by Congo red treatment, where the plate was immersed in 0.1 M NaOH for 15 min to destain. The occurrence of any halo zone around the colonies indicated significant cellulase activity.

For the determination of amylase production, the cultures were streaked onto starch agar medium and incubated for 48 h. Following this, Gram’s iodine was flooded into the culture plates, and excess stain was drained off to observe for clear zones around the colonies (Nithya et al., 2016; Sritongon et al., 2023).

The gelatinase activity of the chosen strains was examined using a gelatin crystal agar medium (Alnahdi, 2012). After incubation for 48 h, the plates were observed for any zone of clearance, using mercuric acid solution. Finally, screening of lipase production was conducted by inoculating the culture into the Tributyrin agar medium. A clear zone around the colonies after 2 days of incubation at 37 °C indicated a positive result (Castaldi et al., 2021).

### 2.6 Compatibility test

The antagonistic activity was assessed to evaluate the coexistence between the two bacterial strains: *Pseudomonas* sp. and *Burkholderia* sp. This assay was designed to further examine the interactions between the bacterial strains chosen for the study (Rangasamy and Saleh, 2025). In the middle of a nutrient agar plate, a single bacterial strain was vertically streaked and then cultured for 24 h at 37 °C. After the specified time period, the other strain was streaked perpendicular to the initial strain. The plate was kept at 37 °C for an additional 24 h, and any inhibition zones between the strains were observed. Nutrient agar plates with no bacterial inoculation were used as controls (Irabor and Mmbaga, 2017; Prasad and Babu, 2017).

### 2.7 Screening of dual culture assay of antagonistic activity

The antagonistic activity of test strains *Pseudomonas* sp. (VITK-1) and *Burkholderia* sp. (VITK-3) was evaluated against selected plant pathogens: *F. oxysporum f.*sp. *lycopersici* and *Ralstonia solanacearum,* which are ordered from the Microbial Type Culture Collection and Gene Bank at the Institute of Microbial Technology, Chandigarh, India (ID: 10270 and 13168). The pathogens were cultured for 7 days at 28 °C in casamino acid-peptone-glucose broth (CPG) and potato dextrose broth (PDB) media using appropriate media, prior to the dual culture assays.

Bacterial strains were standardized to an optical density (OD 600) of 0.6 and streaked 2 cm from one edge of a PDA agar plate, while the fungal culture was streaked 1 cm from the opposite edge on the same medium. Equation 1 was used to establish the inhibition rate after the plates were cultured at 25 °C for 7 days. A completely randomized design was utilized for the triplicate experiment (Rangasamy and Saleh, 2025).


Inhibition rate%=RC−RIRC×100 (1)

Where, RC is the distance from the edge of the fungal colony on control plates.

RI is the distance from the fungal colony to the bacterial inoculum on treated plates.

Antibacterial activity of the endophytic bacteria (*Pseudomonas* sp. and *Burkholderia* sp.) against *R. solanacearum* was assessed using a zone inhibition assay. *R. solanacearum* was cultured overnight on a CPG medium and was standardized to a specific colony-forming unit (10^6^ CFU/mL) concentration. The CPG agar medium was sterilized, poured into plates, and allowed to solidify. A well of 8 mm diameter was cut at the center of the agar plate, and *R. solanacearum* was uniformly spread around the well using sterile cotton swabs. Freshly grown bacterial strains (200 μL) were added to the well, and the plates were incubated at 37 °C for 24 h. By measuring any zone of suppression closest to the well, the antagonistic activity was ascertained (Anith et al., 2021; Madriz-Ordeñana et al., 2022; Mekonnen et al., 2022).

### 2.8 Salt tolerance analysis

Salt tolerance of bacterial strains was tested by inoculating isolates into 10 mL of nutrient broth containing different concentrations of NaCl (0% to 20%). The cultures were then incubated at room temperature for 48 h. Following incubation, samples from each culture were spot-inoculated onto nutrient agar plates to evaluate visible growth and determine the salt tolerance thresholds for the isolates (Fahsi et al., 2021; Fiodor et al., 2023).

### 2.9 Temperature and pH monitoring, and heavy metal tolerance

Minimal salt medium (MSM) broth was used to inoculate isolates to assess bacterial growth under multiple levels of pH (3, 5, 7, 9, and 11) and temperatures (25 °C, 30 °C, and 37 °C), with adjustments made using NaOH and HCl buffers. Absorbance was measured at 600 nm while the culture was incubated at 30 °C with shaking at 150 rpm. Visible growth was also analysed and parameters such as turbidity of the medium and change in colour were also recorded.

Heavy metal tolerance was examined using an MSM medium with varying concentrations of metals (Cd, Co, Zn, Pb, and Fe) ranging from 250 mg/mL to 1,000 mg/mL at 30 °C. Upon visible growth occurred, the concentration was increased to 4,000 mg/mL using 1% inoculum in MSM broth medium at 200 rpm. Subsequently, cultures were spot-inoculated on a nutrient agar medium to observe visible growth, with uninoculated broth serving as a control (Pranaw et al., 2020; Fahsi et al., 2021).

### 2.10 Determination of pathogenicity

The isolates have been streaked onto blood agar and grown for 2 days at 37 °C to assess the hemolytic activity. The plates were then examined for color changes and categorized into three categories: beta (yellowish color), alpha (greenish brown color), and gamma (no visible zone around the colony or color changes in the plate). These categories denote the extent of hematolytic activity of the selected strains (Bhatt and Maheshwari, 2020).

### 2.11 Germination assay, determination of morphological growth, biochemical characterization, and gene expression

#### 2.11.1 Preparation of bacterial suspension

Bacterial isolates were cultured overnight in the nutrient agar medium, and a single isolated colony was transferred into 100 milliliters of nutrient broth in a conical flask using a sterile loop and allowed to grow exponentially at 30 °C. The bacterial culture was centrifuged for 5 min at 1000 rpm following incubation. Upon discarding the supernatant, the cell pellet was rinsed and diluted using a sterile 0.85% saline solution. Sterile saline solution was used to standardize the bacterial count and adjust the suspended bacteria to an OD value of 1 (10^8^ CFU/mL) (Helal et al., 2022).

#### 2.11.2 Tomato seed germination assay on bacterial strains

Commercially purchased tomato seeds were used for the germination assay. The seeds were first surface sterilized by immersing in 70% ethanol for 10 s, followed by surface sterilized treatment with 3% sodium hypochlorite (NaOCl) solution under continuous shaking for 20 min. After sterilization, the seeds were rinsed three times with sterile distilled water (SDW) to remove residual chemicals. Notably, floating seeds were discarded, and the remaining seeds were air-dried under sterile conditions before performing germination. Later on, 15 seeds were soaked for 30 min in 30 mL of individual bacterial suspensions and their consortium (1:1 ratio), each with three biological replicates, while being agitated. After the seeds dried, they were set up on sterile petri plates and placed over the germination sheet. SDW-treated immersed seedlings served as the control. The treatments were designated as follows: T1: un-inoculated control (no bacterial seed treatment), T2: seed treatment with VITK-1, T3: seed treatment with VITK-3, and, T4: seed treatment with VITK-1 + VITK-3 (consortium). Each treatment consisted of 60 seeds with a triplicate for germination. The plates were tightly closed with parafilm to maintain the moisture and were kept for 12 days at room temperature. The following parameters were calculated: germination rate was calculated as the percentage of seeds that germinated by the end of the experiment, seedling vigor index was determined by multiplying the germination percentage by the mean seedling length (seedling shoot + root lengths, measured in cm), relative seed germination calculated by comparing the number of germinated seeds in treated samples with the control, energy of germination was evaluated as the number of seeds germinated within the first 4 days, and daily germination count was recorded by counting the number of newly germinated seeds each day. Detailed formulas are provided in Supplementary Data 1.

Simultaneously, the complete root and shoot length of the seedlings were measured on Day 12. A highly randomized block design was implemented for the experimental setup (Pandey and Gupta, 2020; Fahsi et al., 2021).

#### 2.11.3 Colonization of bacterial strains on tomato seedlings

Previously disinfected and treated seeds were placed in a seedling tray with 90 cavities, each containing two seeds soaked in the respective bacterial suspensions. Once the seedlings emerged, they were watered daily with sterile distilled water. The soil used had an alkaline pH of 7.9, which was pre-tested before the treatment. Seedlings were transplanted into a plastic pot filled with 5 to 7 kg of soil from the agricultural field areas of VIT Brahma Puram. Twenty days after transplantation, treatments were initiated. Ten milliliters of freshly prepared bacterial suspension (OD600nm = 1.0) were administered to the plants once a week. The experiment was conducted in a greenhouse setting at VIT University in Vellore, Tamil Nadu, India, utilizing a completely randomized block design with three separate trials. The greenhouse maintained the humidity between 60% and 75% and the average temperature of 25 °C. At the end of the experiment, chlorophyll content, photosynthesis, various morphological parameters, and biochemical characteristics were measured. Chlorophyll content was assessed using a SPAD meter. The average plant height, thickness of stem, total amount of leaves, number of branches, length of leaf, and diameter were also recorded. Additionally, the fresh and dry weight of the roots and shoots was measured after rinsing the plants with tap water to wash away soil from them.

Subsequently, to determine the nutrient content, including micro and macronutrients, plant samples were then dried in a hot air oven for 3 days at 60 °C. The National Agro Foundation (NAF, Tharamani, Chennai) conducted this analysis (Cottenie, 1980).

#### 2.11.4 Soil analysis and characterization

Soil samples were analysed and characterized by the National Agro Foundations (NAF, Chennai) to better understand the chemical properties, pH, electrical conductivity, and nutrient availability of macronutrients and micronutrients. Characterization of soil is generally performed before the transplantation of seedlings from pro tray to pot (Cottenie, 1980).

#### 2.11.5 Chlorophyll pigmentation

Fresh leaf samples (0.2 g) were obtained and was homogenized with 80% chilled acetone, following the protocol described (Rajalakshmi and Banu, 2015) with slight modification. At 4 °C, the homogenized samples were centrifuged for 10 min at 5000 rpm. After transferring the supernatant to a test tube, acetone was used as the blank, and a UV–visible spectrophotometer was used to determine the absorbance at wavelengths 663 nm and 645 nm. Chlorophyll a and b were calculated from the sample extraction using the following equations (Supplementary Data 2) to determine the pigmentation of the plant.

#### 2.11.6 Determination of protein contents

Total protein content was estimated using Lowry’s method with slight modifications. Protein was extracted from 0.1 g (100 mg) of fresh leaf samples using PBS buffer at pH 7 and vortexed. The fluorescence of the samples was detected at 660 nm using a microplate reader. A 50 mg of Bovine serum albumin (BSA) stock solution was prepared by dissolving it in 50 mL of distilled water. A graph was then constructed to calculate the concentration of the unknown sample (Sarkar et al., 2020).

#### 2.11.7 Total proline estimation

The Ninhydrin method to evaluate bacterial inoculation by quantifying the total protein concentration of fresh plant fragments (Rakesh et al., 2021). Homogenizing 0.5 g of tissue required 10 mL of 3% sulpho-salicylic acid. A 200-microliter extract was collected from the supernatant after discarding the pellet through centrifugation at 1000 rpm for 4 °C at 10 min. An equal volume (1:1 ratio) of 200-microliter acid ninhydrin and glacial acetic acid was added and mixed by vortexing. The mixture was immediately cooled after incubating at 100 °C for 1 h, resulting in a pinkish color formation. A 400 microliter of toluene was added to the extraction, thoroughly vortexed, and allowed to stand for 10 min in the dark to evaluate. After dark incubation, two layers were formed: the top layer (red colour) was separated clearly to measure the OD value at 520 nm in a 96-well microtiter plate. To calculate the proline concentration in the known sample, a standard graph was plotted with toluene serving as a blank and L-proline as a reference.

#### 2.11.8 Carbohydrate estimation

The phenol-sulfuric technique was employed to quantify the amount of carbohydrates in treated PGPR seedlings (Mohebi et al., 2018). After transferring fresh tissue samples to a boiling test tube and grinding them with five milliliters of 2.5 N HCl, they were incubated at 100 °C. Following incubation, the reaction mixture was subsequently supplemented with a 96% sulfuric acid and sodium carbonate solution. A Thermo Fisher Scientific microtiter plate reader was used to detect absorbance at 490 nm, with all test reagents except the plant extract used as blanks. To ascertain the samples’ unknown carbohydrate content, glucose was selected as the standard reference.

#### 2.11.9 Plant extraction and determination of antioxidant activity

Plant samples (0.4 g) were dried at 60 °C for 2 days. The dried samples were mixed with 10 mL of methanol (HPLC grade) in a conical flask. The flask was covered with aluminum foil to prevent evaporation, and the mixture was vortexed overnight. The sonication technique was applied to enhance agitation, and the mixture was then passed through the filter paper made by Whatman No.1. The extracted sample was dried, resuspended in methanol to form a stock solution, and stored at −20 °C for further analysis.

Antioxidant activity was evaluated using the DPPH (1,1, diphenyl-2picrylhydrazyl) assay with slight modifications (Phuyal et al., 2020). The concentration of the plant extract was diluted with methanol to a range of 20 mg/L – 100 mg/L. A standard L-ascorbic acid stock solution was used for comparison. The different concentrations of plant extract were mixed with a 0.004% DPPH solution (dissolved in methanol) and incubated for 30 min at room temperature. Methanol and DPPH were used as blank and control, respectively. Following incubation, the antioxidant activity was calculated using the specified formula (Supplementary Data 3), and the absorbance was measured at 517 nm.

#### 2.11.10 Phyto compound production

Two techniques were employed to determine the phenolic and flavonoid content using the Folin–Ciocalteu and aluminum chloride methods, respectively. For phenolic content, 100 microliters of the extracted plant sample (described in section 2.10.9) was mixed with 400 microliters of methanol, followed by the addition of 150 microliters of Folin Ciocalteu reagent and 20% sodium carbonate (Chavan et al., 2013). The solution was vortexed thoroughly and incubated in the dark for 1 h. Methanol and Folin–Ciocalteu reagents were used as blanks. A standard curve was plotted using gallic acid to determine the total phenolic content in the test sample, and absorbance was measured at 650 nm. For flavonoid content, 100 microliters of the test sample were combined with 400 microliters of methanol, followed by the addition of 100 microliters of 10% AlCl_3_ and 1 M sodium acetate (Phuyal et al., 2020; Rakesh et al., 2021). After the mixture was vortexed, and then left for 45 min at room temperature. Using a microtiter plate with methanol alone as the blank (without test samples), absorbance was measured at 415 nm.

#### 2.11.11 Quantification using RT -PCR

The expression of nutrient-regulating genes in *S. lycopersicum* was analyzed using quantitative real-time PCR (qRT-PCR). Gene expression was assessed in plants treated with a consortium of bacterial suspensions. Root samples were collected from four treatment groups and a control group, with biological replicates for each, and were frozen in liquid nitrogen (N_2_). Two hundred milligrams of tomato seedlings’ lateral roots were used in each sample group for RNA extraction using RNA-Isoplus (Takara), and Nanodrop (Thermo Scientific) was used to quantify the yield. To convert the extracted RNA into cDNA, reverse transcription was performed using a cDNA synthesis kit (HiMedia). On a Bio-Rad (USA), real-time PCR amplification was carried out with a Takara SYBR master mix. Briefly, the amplification reaction mixture contained a total volume of 10 μL, comprising 2 μL of both forward and reverse primers, 2 μL of nuclease-free water, 5 μL of the reaction mixture, and 1 μL of a template. The qRT-PCR conditions were set as follows: initial denaturation at 95 °C for 10 min, followed by 40 cycles of 95 °C for 15 s, 56 °C for 10s, and 72 °C for 15 s. This was followed by a melting curve stage of 95 °C for 10 s and 60 °C for 1 min, with a gradient PCR set from 55 °C to 62 °C. Primers were designed using Oligo 7 software (Supplementary Table 1) and were based on the nutrient expression gene sequence of *S. lycopersicum* obtained from BLAST and NCBI. For *S. lycopersicum*, the actin gene served as the housekeeping gene (Saia et al., 2015).

### 2.12 Statistical analysis

All the tests and experiments described in this study were carried out in triplicate, and the results were expressed as mean ± standard deviation (SD), calculated using Microsoft Excel. Graphical data was generated using GraphPad Prism and JMP Pro 17, and Duncan’s multiple range test was also performed. The experimental design adopted here followed a completely randomized design. Significance differences among treatments were calculated using one-way ANOVA, followed by a two-tailed Student’s *t*-test and Duncan’s multiple range test for comparisons.

## 3 Results

### 3.1 Isolation and molecular characterization of PGPR

**Figure 2:**
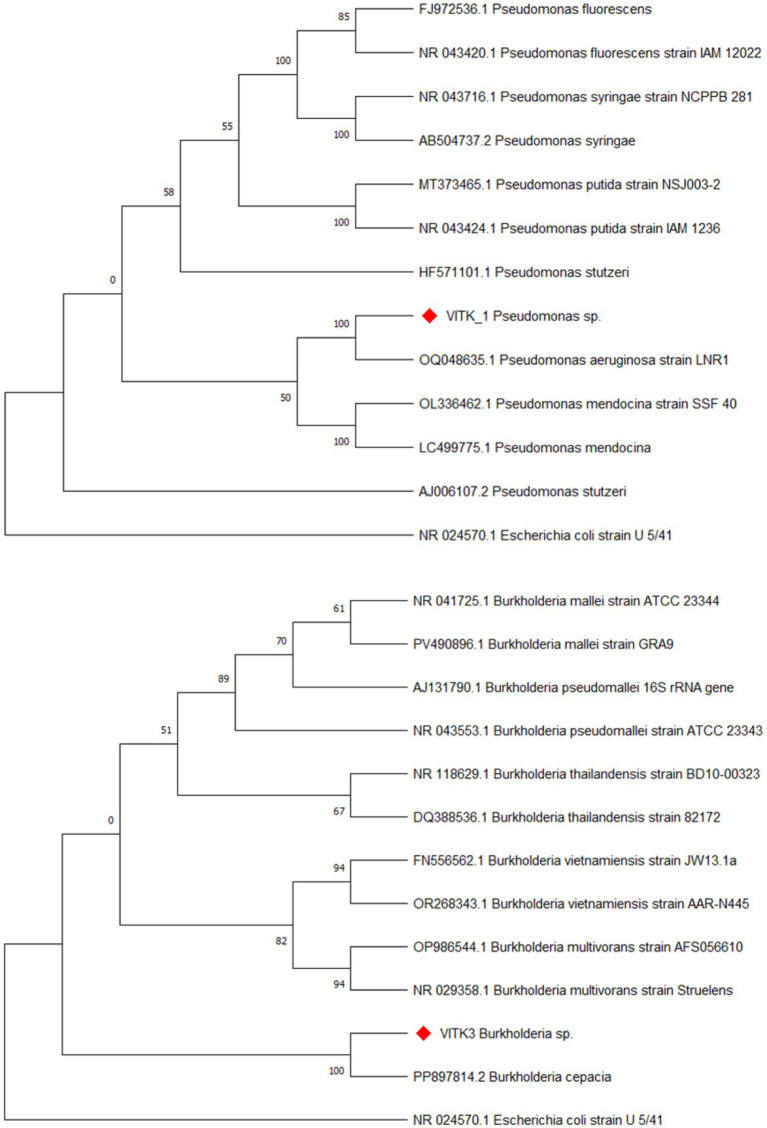
The phylogenetic tree shows the evolutionary relationships of *Pseudomonas* sp. (VITK-1), *Burkholderia* sp. (VITK-3), and related reference sequences retrieved from GenBank based on 16S rRNA gene sequences. The tree was constructed using the Neighbour-Joining method in MEGA11 with evolutionary distances calculated by the maximum composite likelihood method. Bootstrap analysis was performed with 1,000 replicates, and bootstrap values ≥50% are illustrated at the branch nodes, and *Escherichia coli* strain U 5/41 was used as the outgroup.

The bacterial isolates VITK-1 and VITK-3 were obtained from the VIT agriculture field and cultured on the nutrient agar medium using the quadrant streak method. Detailed colony characterization and morphological analysis revealed that VITK-1 appeared to be dull-white in colour with a round, entire margin, and slight elevation. The colonies were opaque in nature, had a notable bluish-green tint in the vicinities of streak lines, and were small to medium-sized. Whereas, colonies of the strain VITK-3 have a similar size as compared to VITK-1, but were white/creamy in color round, marginated and non-transparent. Supplementary Figure 1C illustrates the scanning electron microscopy (SEM) analysis, which revealed that both strains had a rod/coccoid shape and were Gram-negative. Figure 2 shows the 16S rRNA sequencing and BLAST identification. VITK-1 was identified to be *Pseudomonas* sp. with a similarity index of 99%, and the strain VITK-3 was identified to be *Burkholderia* sp. with a similarity index of 99.5%. The GenBank accession numbers assigned to these sequences were OP102696 and PP897814, respectively.

### 3.2 Determination of plant growth promotion traits and enzymatic activity

The qualitative assessment of phosphate solubilization by the isolated bacterial strains was performed using NBRIP agar medium. Following a 48-h incubation period, both strains produced clear zones around the colonies (Supplementary Figure 1D), indicating positive phosphate solubilization activity. Among the two strains, VITK-1 shows the largest zone, suggesting higher phosphate solubilization efficiency as compared to VITK-3. The comparative results are summarized in Supplementary Table 2. Indole-3-acetic acid (IAA) production was evaluated using the tube test method in the presence of tryptophan. As shown in Supplementary Figure 2, VITK-3 exhibited significantly higher IAA production after 48 h of incubation at 37 °C in nutrient broth, whereas VITK-1 produced a comparatively lower amount of IAA.

The siderophore production was assessed to determine the iron-chelating ability of the isolates VITK-1 and VITK-3. Both strains exhibited clear zones enveloping the colonies, which were indicated by a yellowish-orange color and are comparable to the positive solubilization of siderophore formation as listed in Supplementary Table 2 and Supplementary Figure 1E. The growth of selected strains in nutrient media in a test tube after 24 h of incubation as shown in Supplementary Figure 1F. From the results, both strains exhibited a significant amount of ammonia production, as indicated by the orange color formation when a small amount of Nessler’s reagent was added. This color change confirmed a positive response for ammonia production.

As shown in Supplementary Table 2, qualitative screening of nitrogen fixation was performed on Jensen’s medium following a 24-h incubation period. Both VITK-3 and VITK-1 showed growth on the plate (Supplementary Figures 1H,I), which constitutes an obvious indication of a beneficial result. As a comparable screening technique for the production of hydrogen cyanide, on the nutrient agar plate that contains sodium carbonate and picric acid. Supplementary Table 2 shows the production of hydrogen cyanide. When compared to VITK-3, the reddish-orange vibrant color that emerged from the VITK-1 strains (Supplementary Figure 1G) revealed the positive response, as evidenced by the absence of notable color changes as a negative result.

As indicated in Supplementary Table 2, after 48 h of incubation, VITK-1 and VITK-3 showed a particularly significant difference in growth on skim milk agar, with a noticeable halo zone circling the colonies. On gelatin crystal agar, the isolates VITK-1 and VITK-3 demonstrated a positive gelatinase activity. After being dissolved in a solution of mercuric acid, the isolates’ growth was assessed by the development of a halo zone surrounding colonies. VITK-1 showed prominent yellowish-white zones encircling the streaking area. The cellular activity is comparable to that of VITK-1, as confirmed by VITK-3. Supplementary Table 2 indicates that both outcomes have a positive reaction. During Gram’s iodine staining, the VITK-1 revealed a massive clear zone surrounding the colonies. After 48 h of incubation, VITK-3 exhibited minimal zone around the colonies, suggesting slow enzyme activity. After 48 h of incubation, VITK-1 and VITK-3 were seen to be present in a distinct and vigorous zone surrounding the colonies. These findings suggest that the enzyme hydrolyzes by generating lipase activity, as listed in Supplementary Table 2.

### 3.3 Antagonistic compatibility analysis

**Figure 3:**
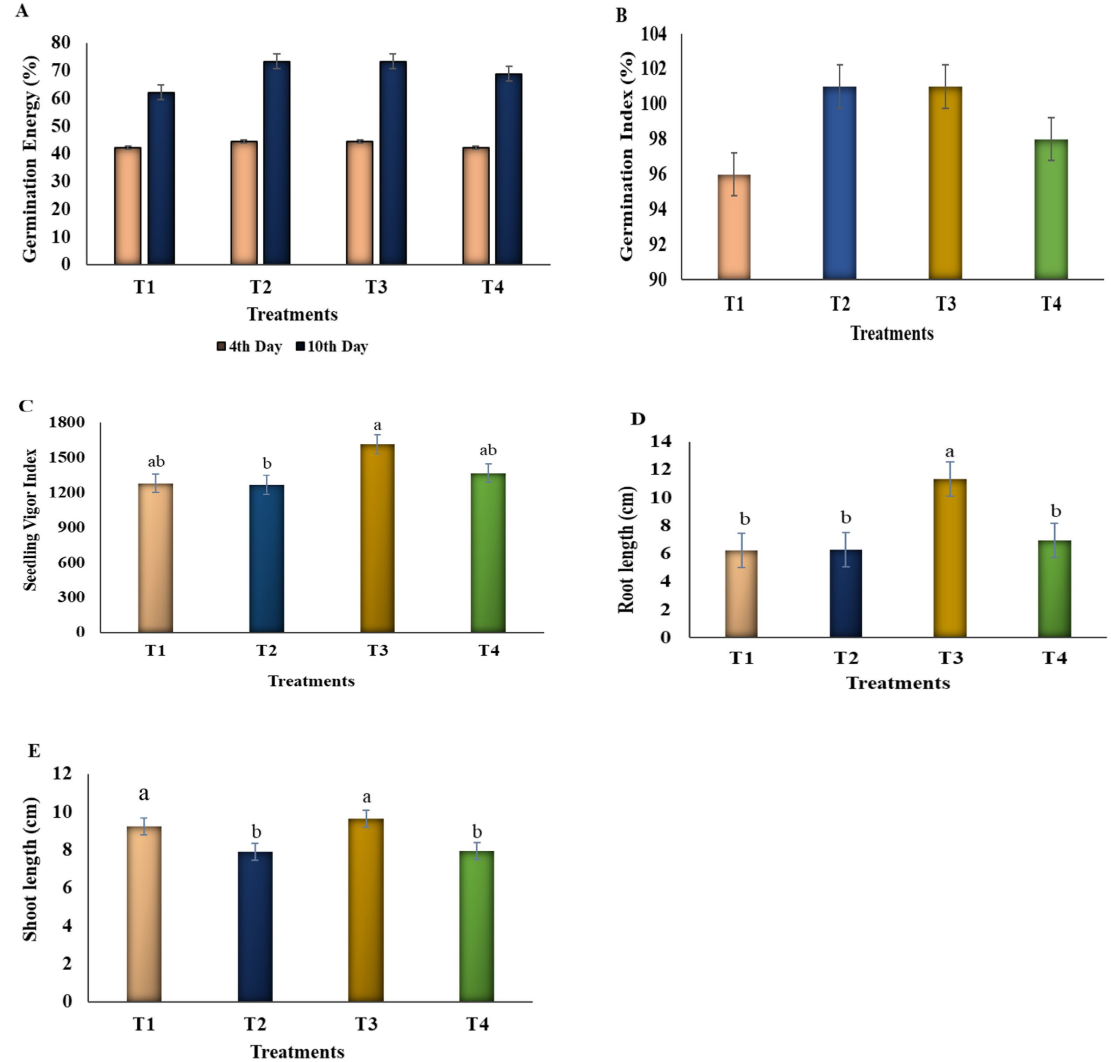
The effect of plant growth-promoting rhizobacteria (PGPR) on seed priming for germination assay in the early growth of tomato seedlings. For the initial priming, seeds per treatment were soaked in bacterial suspension (10^8^ CFU/mL) for 30 min with the designed treatment conditions: individual strains (*Pseudomonas* sp. VITK-1; T2), VITK-3 (*Burkholderia* sp. VITK-3; T3), and their consortium VITK-1 + VITK-3 (1:1 ratio; T4). The control was served with sterile-distilled water (T1). Seeds were germinated on sterile petri plates and maintained in moist conditions for 12 days at room temperature. **(A)** Germination energy, **(B)** Germination index, **(C)** Seedling vigor index, **(D)** Root length, **(E)** Shoot length. Data represents the error bar of mean and standard error along with three biological replicates (One-way ANOVA *p* < 0.05, *p* < 0.001, *p* < 0.0001). Different letters represent the statistical significance, respectively, in treatments (T2, T3, and T4) and control groups according to the student *t*-test.

Supplementary Table 3, which shows the growth inhibition patterns of VITK-1 and VITK-3, displays the findings of the antagonistic activity study. The extent of the inhibition observed reflects the level of compatibility or antagonism between the isolates. Based on the findings, *Pseudomonas* sp. and *Burkholderia* sp. successfully coexisted on the same nutrient agar plate without inhibiting each other’s growth after a 24-h incubation period. The absence of antagonistic interactions confirms a positive compatibility result, as illustrated in Figures 3A–C using scanning electron microscopy.

#### 3.3.1 Antagonistic activity assay

In the dual culture assay, the strains VITK-1 and VITK-3 inhibited the growth of test pathogens, including bacterial and fungal pathogens. *These included R. solanacearum, followed by F. oxysporum f.*sp. *lycopersici* was confirmed through the observations. Quantitative measurements revealed that VITK-1 exhibited the highest growth inhibition rate against *F. oxysporum f.*sp. *lycopersici* (76.5%) in PDA growth medium, followed by *R. solanacearum* (71.8%). Similarly, the bacterium VITK-3 showed inhibition of mycelia growth and growth suppression of the gram-negative bacterium *R. solanacearum* at 35.7%, demonstrating antagonistic activity against fungal pathogens. Supplementary Table 3 summarizes the lowest inhibition rate, 64.1%, against *F. oxysporum f.*sp. *lycopersici*. The findings suggest that the isolates synergistically inhibit the pathogen.

### 3.4 Stress tolerance (salt stress tolerance)

Strain VITK3 exhibited remarkable salt tolerance, thriving even in concentrations as high as 20% NaCl when assessed using the spot-inoculation method. Conversely, VITK1 displayed slightly lower tolerance, with growth observed up to 15% salinity stress (Supplementary Table 3). The bacterial strains were observed under various pH and temperature conditions. They showed a maximum tolerance range of pH at 3 to 11, with a preference for 37 °C. Minimal growth occurred at pH 4, with the maximum range at pH 9.

Heavy metal resistance testing revealed that VITK-1 exhibited the highest resistance, followed by the other isolates (Supplementary Table 4). The specific order of heavy metal resistance among the isolates is as follows: Fe > Cd > Co > Pb > Zn.

### 3.5 Pathogenicity testing

According to Supplementary Table 3, these findings showed no indication of hemolytic activity on blood agar media. From the observations made after the stipulated incubation period, both strains failed to exhibit any hemolytic activity under the given conditions.

### 3.6 Plant growth assay and biochemical traits

#### 3.6.1 Tomato seed germination

Over 12 days, the bacterial treatments (T1, T2, and T3) and the bacterial consortium (T4) had a significant impact on tomato seed germination Figure 3. Results indicated that treated seeds showed a notable increase in germination efficiency compared to control groups, as observed from Supplementary Figure 3A. However, there was no statistically significant change in germination percentage between treated seeds and the control (Supplementary Figure 3B). Among the treatments, single inoculum treatments (T2: T3) resulted in the most pronounced improvements, with germination energy increased by 73.3%, germination index by 101%, and enhanced root and shoot length compared to other treatments (Figure 3; Supplementary Figure 4).

#### 3.6.2 Growth attributes

**Figure 4:**
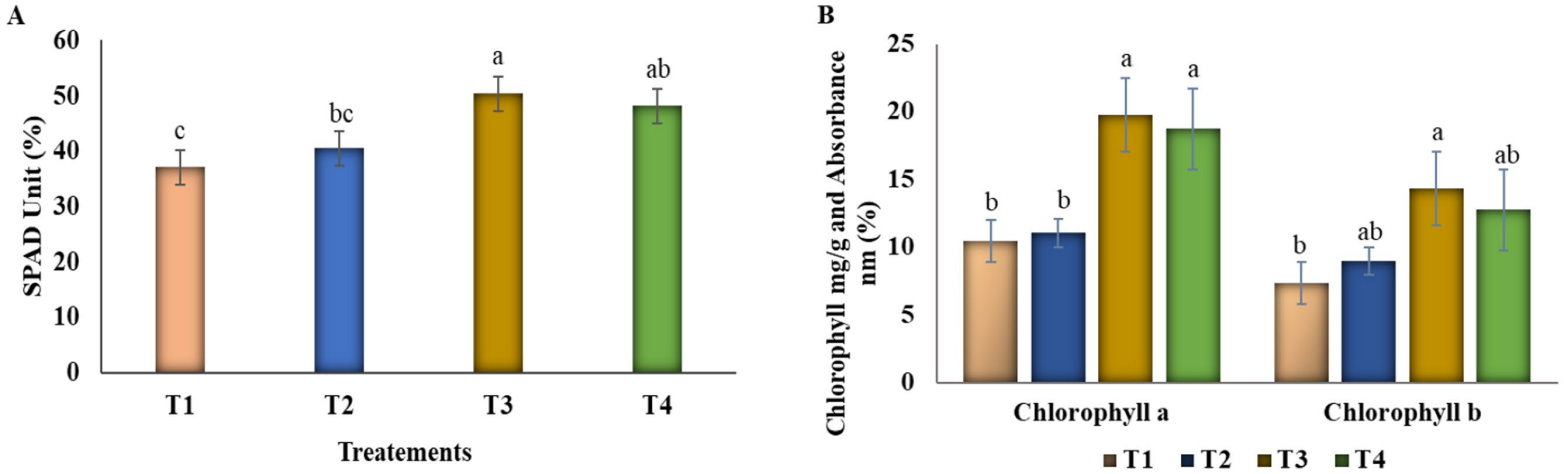
Effect of bacterial seed priming on chlorophyll content and SPAD values of tomato plants under *in vivo* conditions after transplantation. Tomato seedlings raised from seeds primed with bacterial suspensions were transplanted into greenhouse conditions. The leaf was measured in the early morning using a SPAD meter, and chlorophyll a and b contents were quantified from fresh leaf samples. **(A)** SPAD units and **(B)** chlorophyll a and b content of tomato plants were increased under different treatment condition T2:T3:T4 compared with the control treatment. Data represents the error bar of the mean and standard error along with three biological replicates. Different letters show a statistical significance among the treatments at *p* < 0.05 according to Duncan’s multiple comparison test.

The bacterial treatments, including two individual strains and one bacterial consortium, improved plant growth and height in tomato seedlings (graphical experiments, Supplementary Figure 5). Soil tests were analyzed before transplanting, and the report is mentioned in Table 1. Although there was no discernible difference between the two individual treatments, the bacterial consortium enhanced plant height, root elongation, and stem diameter in comparison to the uninoculated control. The combination of VITK-1 and VITK-3 showed the most notable improvement in plant height, root length, and, a 50.3% rise in the SPAD range (Figure 4A), along with enhancement in other physiological parameters (Table 2; Supplementary Figure 6). Root samples from inoculated plants showed elevated levels of certain nutrients, such as zinc, nitrogen, and iron, particularly in the T2 and T3 treatments (1.58 to 1.68 mg), followed by the consortium (1.70 mg), (Table 3). Bacterial treatments boosted the overall nutrient content in root samples, with the single inoculum treatments displaying clear variations in nutrient levels compared to the control under alkaline soil conditions.

**Table 1:** Characterization of soil (VIT agriculture field).

S. No.	Parameters	Unit	Results
1	pH	—	7.70–7.9
2	Electrical conductivity	ms/cm	0.210
3	Organic matter	%	1.08
4	Nitrate nitrogen	mg/kg	26.1
5	Available phosphorus	mg/kg	15.81
6	Potassium exchangeable K	mg/kg	191
7	Calcium exchangeable Ca	mg/kg	1728
8	Magnesium exchangeable Mg	mg/kg	390
9	Sodium exchangeable Na	mg/kg	285
10	Sulfur available S	mg/kg	8.4
11	Zinc available Zn	mg/kg	0.58
12	Manganese available Mn	mg/kg	10.31
13	Iron available Fe	mg/kg	15.48
14	Copper available Cu	mg/kg	2.00
15	Boron available B	mg/kg	0.4
16	Cation exchange capacity	meq/100 g	13.62
17	K saturation	%	3.60
18	Ca saturation	%	63.44
19	Mg saturation	%	23.86
20	Na saturation	%	9.10

**Table 2:** Effect of bacterial inoculation on physiological growth parameters of tomato plants under alkaline soil conditions.

Parameter	Control (T1)	VITK-1 (T2)	VITK-3 (T3)	VITK-1 + VITK-3 (T4)
Plant height (cm)	78.5 ± 7.23	81 ± 6.68^ab^	88.5 ± 5.74^a^	80 ± 3.74^ab^
Root height (cm)	28.72 ± 2.67	31 ± 5.597^ab^	36.82 ± 6.34^a^	35.55 ± 4.39^ab^
Fresh weight (mg)	81 ± 6.37	80.5 ± 4.43^a^	88.75 ± 4.5^a^	83.25 ± 7.85^a^
Dry weight (mg)	18.12 ± 2.78	18.87 ± 3.32^a^	20.1 ± 7.16^a^	19 ± 3.13^a^
Fresh root weight (mg)	7.07 ± 0.48	9.40 ± 0.39^a^	9.60 ± 0.58^a^	8.09 ± 0.54^b^
Dry root weight (mg)	3.93 ± 2.41	5.37 ± 3.59^a^	6.18 ± 2.96^a^	5.13 ± 2.43^a^
Stem diameter (cm)	3.1 ± 0.38	3.32 ± 0.04^a^	3.35 ± 0.26^a^	3.02 ± 0.1^a^

The table represents plant height, root length, fresh and dry shoot weight, fresh and dry root weight, and stem diameter for plants in an experiment with the following treatments: control (uninoculated), VITK-1, VITK-3, and a consortium of VITK-1 + VITK-3. Data table expressed as mean ± standard deviation (SD). Treatments T2 (VITK-1), T3 (VITK-3), and T4 (VITK-1 + VITK-3) are compared with the control. Different letters indicate statistically significant differences among treatments, according to the Student’s *t*-test, and a comparison test was performed by Duncan’s multiple test *p* < 0.05, respectively.

**Table 3:** Efficacy of bacterial treatment on nutrient accumulation in dried root samples of plants grown in alkaline soil.

Parameters	Control (T1) (mg)	VITK-1 (T2) (mg)	VITK-3 (T3) (mg)	VITK-1 + VITK-3 (T4) (mg)
Nitrogen (N)	1.39	1.58	1.68	1.70
Phosphorus (P)	0.22	0.23	0.30	0.38
Potassium (K)	0.97	0.99	1.02	0.79
Calcium (Ca)	0.78	0.79	0.74	0.91
Magnesium (Mg)	0.30	0.22	0.23	0.19
Sodium (Na)	0.42	0.47	0.44	0.39
Zinc (Zn)	30.16	35.87	37.47	32.98
Iron (Fe)	1579.62	2144.12	1689.04	1667.19
Manganese (Mn)	92.94	42.11	28.54	26.33
Copper (Cu)	31.67	37.61	36.67	34.45

Roots were collected, dried at 60 °C in a hot air oven, and analyzed for nitrogen (N), phosphorus (P), potassium (K), calcium (Ca), magnesium (Mg), sodium (Na), Zinc (Zn), iron (Fe), manganese (Mn), and copper (Cu) to evaluate the effect of bacterial application in enhancing nutrient uptake.

#### 3.6.3 Analysis of plant growth chlorophyll content

Total chlorophyll content increased in all treatments, including bacterial treatments applied to alkaline soil stress conditions (pH 7.9), as illustrated in Figure 4B. Notable and significant differences in chlorophyll content were observed in bacterial-treated plants compared to the untreated control group. Significant variation was observed in both total chlorophyll a and b content. Among the treatments, chlorophyll a concentration was significantly higher in seedlings treated with T3, which showed a 68% increase, and T2, with a 48.2% increase, and consortia treatment T4, which showed a 65% increase. Similarly, chlorophyll b content also showed a similar enhancement in these treatments, with T3 showing a 66% increase, followed by T2, with a 38% increase, and T4, at 60.5%, exhibiting higher values.

#### 3.6.4 Estimation of protein, proline, and carbohydrate content

**Figure 5:**
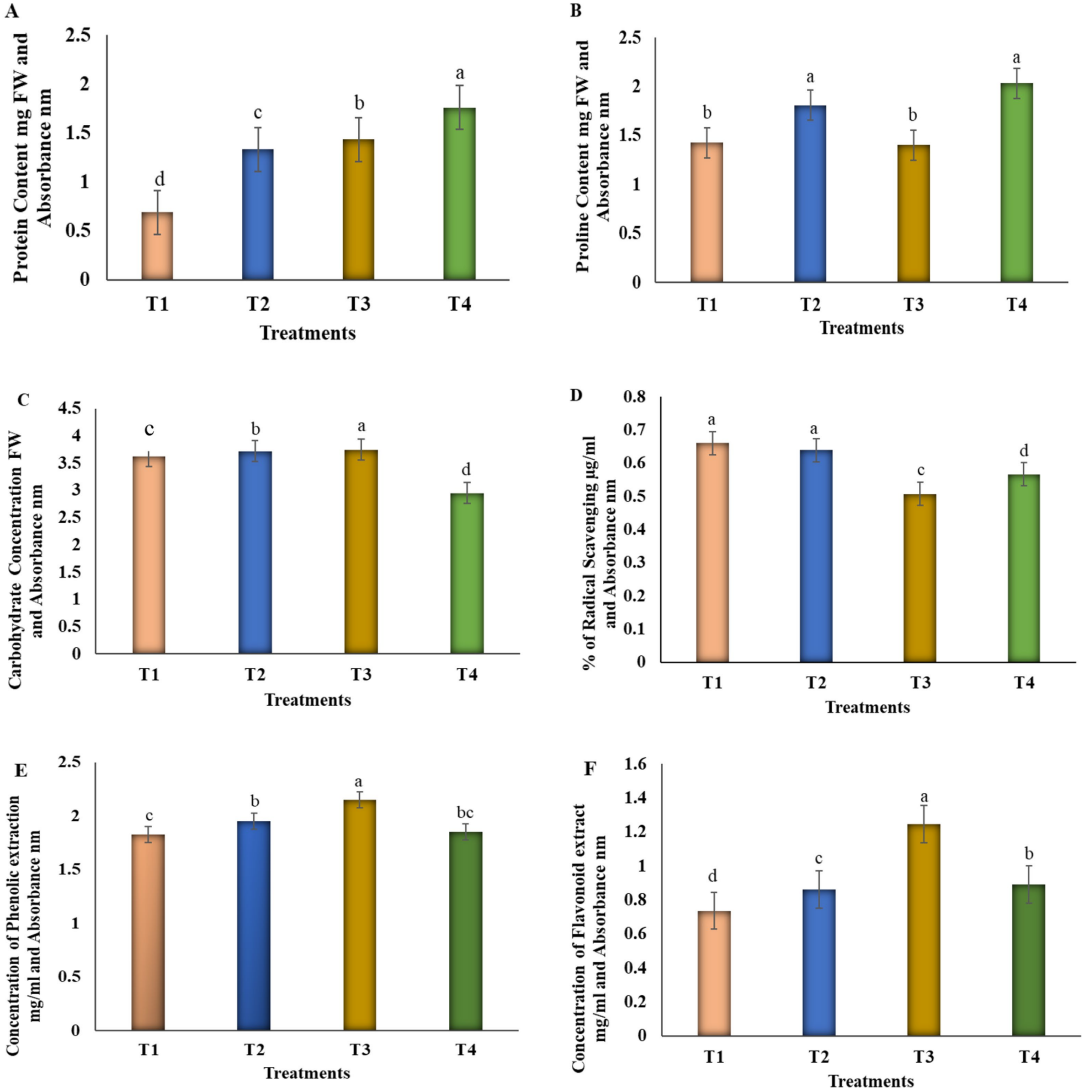
The impact of *Pseudomonas* sp.*, Burkholderia* sp., and their combined inoculation on the growth and biochemical characterization of tomato plants grown under alkaline soil conditions. Tomato seedlings were transplanted into alkaline soil, and bacterial suspension treatments were initiated 10 days after transplantation by root drenching. Plants were supplied with individual bacterial inoculants and a consortium treatment, while distilled water was the control. Bacterial treatments were applied once per week, and plants were maintained under controlled temperature and humidity conditions. Plant growth and biochemical changes were evaluated following bacterial treatments. **(A)** Protein content, which was significantly higher in the consortium treatment (T4) suggesting enhanced metabolic activity; **(B,C)** proline and carbohydrate content, which were elevated in both T4 (69.5%) and T2 (66%) treatments, indicating increased tolerance under alkaline soil conditions; **(D)** % of radical scavenging activity, T2 (63.97%), indicating increased antioxidant defense; and **(E,F)** phenolic and flavonoid contents, higher at T4 treatment compared with other treatment groups, which demonstrating stress mitigation. Different letters in the alphabet imply statistical significance among the bacterial treatments (T2, T3, and T4) compared to the control groups, respectively, according to the Student *t*-test. The error bar represents the mean and standard error of the one-way ANOVA test; *p* < 0.005, *p* < 0.001, and *p* < 0.0001.

Figure 5A illustrates the results from the estimation of total protein content under alkaline stress conditions. Protein content was higher on T4 at a 54.5% increase in consortia treatment, according to the results comparing individual and consortia treatment under stress conditions. Among other treatments, seedlings treated with T3 displayed a substantially greater protein content (50%) than those treated with T2, which showed a 45.2% increase. These findings imply that seedlings treated with consortia had a higher protein content than those treated with other treatments and the control (untreated) group.

The ninhydrin and phenol-sulfuric acid techniques were used to evaluate the proline and carbohydrate contents of fresh leaf samples. When the bacterial treatments were exposed under alkaline stress conditions, the total proline and carbohydrate content increased in a comparable study. The proline content of T4 was the most significant of the four treatments at 69.5%, followed by T2 at 65% and T3 at 58%. In T4 and T2, proline activity was higher. There was an increase in proline activity on the bacterial treatments as compared to the control groups, as shown in Figure 5B. Bacterial treatment under alkaline stress induced T4 to increase by 45%, T2 to increase by 66%, and T3 to exhibit the same amount of carbohydrate activity. Figure 5C shows that under alkaline stress, there was a significant difference in soluble sugar between the treatment and control groups in the comparable research (*p* < 0.001).

#### 3.6.5 Antioxidant activity, phenolic and flavonoid content

The antioxidant activity was assessed based on the absorbance of color formation, which is directly proportional to the concentration of free radicals. Under stress conditions, lower concentrations of antioxidants were observed in T3, with a reduction of 50.75%, followed by T4 at 56.70%, as illustrated in Figure 5D. Among the treatments, T2 demonstrated higher enzymatic activity, increasing by 63.97% in leaf extract, followed by the control treatment, which showed 66.01% higher antioxidant activity under stress conditions. Phenolic and flavonoid contents were observed under bacterial inoculation and compared with the control group, as illustrated in Figures 5E,F. In seedlings, inoculation with bacterial treatments VITK-1 and VITK-3 in the consortium (T4) exhibited the highest phenolic content at 60.9% and flavonoid content at 52.5%, followed by individual treatment T3, which ranged from 71.9% to 65.7%, and T2, which ranged from 68.2% to 58.3% in both phenolic and flavonoid content when compared with control group showed significant variations. These results suggest that bacterial inoculation treatments significantly influenced phenolic and flavonoid production, with marked differences observed among the groups under stress (*p* < 0.05, *p* < 0.001).

#### 3.6.6 Nutrient and stress-regulating gene expression analysis

**Figure 6:**
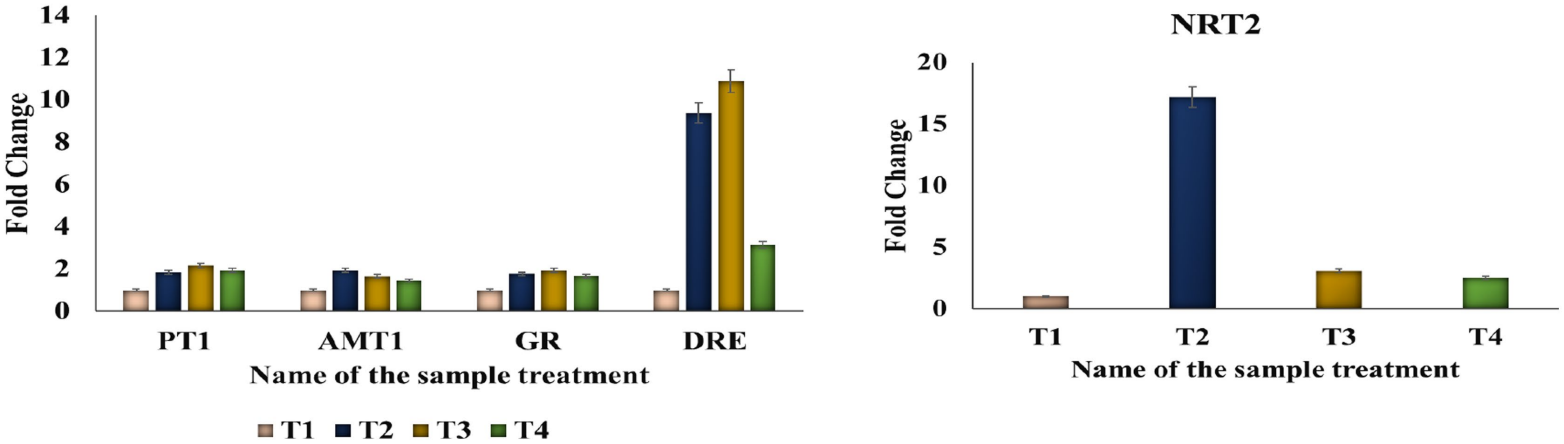
Relative expression of nutrient transporter and stress-responsive genes in tomato seedlings’ roots under alkaline soil conditions following bacterial inoculation. Bar graphs show the relative gene expression levels of *PT1* (phosphate transporter-1), *AMT1* (ammonium transporter-1), *NRT2* (nitrate transporter), *GR* (glutathione reductase), and *DRE* (dehydration-responsive element) in tomato roots treated with individual bacterial inoculants and a bacterial consortium, compared with untreated control plants. Gene expression was quantified under alkaline stress conditions. *PT1* expression was significantly upregulated in consortium-treated plants (fold change 5.38), indicating enhanced phosphate mobilization. Increased expression of *AMT1* and *NRT2* suggests improved nitrogen acquisition through ammonium and nitrate uptake, with a fold change of 1.66 and 2.51. Upregulation of *GR* and *DRE* reflects enhanced stress tolerance and reactive oxygen species scavenging, indicating improved plant survival mediated by plant growth-promoting rhizobacteria (PGPR) under alkaline soil stress.

The expression of three nutrient-regulating (*NRT2*, *PT1*, and *AMT1*) and two stress response genes (*GR* and *DRE*) was analyzed in treated plant roots under alkaline soil conditions using a student’s *t*-test (Figure 6). This experiment assessed the ability of bacterial treatments to protect seedlings from stress conditions. All the genes were upregulated in treatments T2:T3:T4, with fold changes ranging from 5.19, 1.95, and 5.38 for *NRT2*, *GR*, and *DRE*, respectively. Among the genes, *PT1* and *AMT1* had fold changes ranging from 1.95 to 1.66. In the consortium treatment, *NRT2* gene expression showed an elevated upregulation with a fold change of 2.51, and *GR* had a fold change of 1.69. Notably, the consortium treatment significantly upregulates the expression of *DRE*, *PT1*, and *AMT1* compared to the control group, as illustrated in Figure 6.

## 4 Discussion

The present study investigated the role of bacterial consortia in alkaline soil, focusing on their impact on nutrient uptake through tomato roots and gene expression regulation with greenhouse experiments. High stress conditions are a major abiotic factor that limits nutrient availability and affects plant growth (Egamberdieva et al., 2019; Xu et al., 2023). The observed variations in physiological and molecular pathways not only enhanced plant growth and development but also contributed to improved alkaline stress tolerance by modulating intercellular mechanisms (Yang et al., 2024). Treatment with bacterial consortia and individual strains was conducted in triplicate at an alkaline pH of 7.9–8.5 (Dixit et al., 2020), significantly increasing the initial germination rate, vigor index, and root length, consistent with previous studies (Suárez-Moreno et al., 2012; Nakahara et al., 2022; Singh et al., 2023a). The notable enhancement in seed germination rate, early root hair elongation, and initiation of leaf physiological processes facilitates improved regulation of reactive oxygen species (ROS) and nutrient uptake under alkaline soil stress (Liu et al., 2022). In addition to improving plant biomass accumulation, early vigor, and yield potential, these early-stage enhancements exposed to alkaline conditions reduce osmotic imbalance (Yang et al., 2024). Although studies on the T3 endophytic bacterium under stress conditions are limited, our findings suggest that its treatment significantly improved tomato seedling growth compared to the control group (Hwang et al., 2025). The treatment of individual strain (T2) and bacterial consortia further enhanced root length and total seedling growth, partially aligning with previous reports on germination percentage (Khatun et al., 2018). Many PGPR isolates have been shown to promote plant growth and development in greenhouse studies; however, research on their application in alkaline soils, particularly using bacterial consortia, remains limited. The consortia treatment exhibited a significant increase in overall physiological parameters compared to individual treatments and control groups, showing a highly significant difference. Additionally, an increase in root biomass was observed under saline stress conditions. Such increases in root biomass are associated with physiological changes under stress conditions, particularly enhanced root-mediated water transport and improved regulation of osmotic balance and nutrient uptake. These responses are commonly associated with stress-induced enzyme activity, which supports plant defense mechanisms and promotes shoot and root growth under stressful environmental conditions (Hu et al., 2024). The application of PGPR, including *Pseudomonas* and *Burkholderia* species, has been previously reported to enhance plant growth by solubilizing organic and inorganic nutrients in the rhizosphere (Ahemad and Khan, 2010; Gao et al., 2015; Nakahara et al., 2022). These findings highlight how PGPR may be used to improve soil health and increase agricultural yields when plants are exposed to stress.

PGPR contributes to plant growth in challenging soil environments through various mechanisms to enhance stress tolerance and adapt to pH with their PGPR traits including IAA, cyanide, siderophore, phosphate solubilization, nitrogen-fixing and ammonia (Egamberdieva et al., 2019). The strains VITK-1 and VITK-3 were found to facilitate mineral adsorption, particularly phosphate solubilization, and regulate root exudates for IAA production. This process plays a crucial role in promoting phytohormone activity by releasing secondary metabolites (Mittal et al., 2008; dos Santos et al., 2021; Kalyandurg et al., 2022). IAA synthesis is particularly important for root growth, as it aids host plants in recruiting endophytic bacteria under both low and high pH conditions. The bacteria can produce PGPR traits more effectively when exposed to moderate-to-high environmental stress conditions, further supporting plant adaptation and growth (Xie et al., 1996; dos Santos et al., 2021; Ganesh et al., 2024). Another critical function of PGPR is siderophore production, which chelates iron to enhance host nutrient uptake, facilitating chlorophyll and chloroplast biosynthesis (Sultana et al., 2021). In addition, PGPR produces hydrogen cyanide, ammonia, and nitrogen-fixing compounds, all of which are essential to increasing nitrate’s bioavailability in the rhizosphere. Hydrogen cyanide production by these strains also contributes to plant protection by degrading pathogen cell walls and stimulating defense mechanisms against both biotic and abiotic stress conditions (Pandey and Gupta, 2020b).

In addition to previous reports, our findings suggest that *Pseudomonas* sp. VITK-1 and *Burkholderia* sp. VITK-3 act as biocontrol agents against plant pathogens such as *F. oxysporum f.*sp. *lycopersici* and *R. solanacearum*. These strains share characteristics with other closely related species, including *P. aeruginosa FG106* and *B. ambifaria* (Simonetti et al., 2018; Kalyandurg et al., 2022), which have been reported to inhibit various pathogens, such as *Alternaria alternata*, *Botrytis cinerea*, *Clavibacter michiganensis*, *Phytophthora colocasiae*, and *Xanthomonas euvesicatoria pv. Perforans*. Although bacterial biofilm production increases the bacteria’s ability to survive under stress, it has no direct effect on plant protection.

*In vitro* studies revealed that VITK-1 and VITK-3 produce hydrolytic enzymes such as cellulase, protease, lipase, and amylase, which directly inhibit phytopathogens by degrading or weakening fungal cell walls and indirectly enhance nutrient accumulation through improved root metabolic activity, thereby promoting plant growth and development rather than activating defence mechanisms. Additionally, by strengthening the plant’s defenses, these strains promote systemic resistance and aid in the management of disease (Palumbo et al., 2007; Nithya et al., 2016; Jadhav et al., 2017; Barrera-Galicia et al., 2021; Kalyandurg et al., 2022). Notably, Saline-alkaline stress conditions are often interconnected, leading to oxidative damage in host crops while simultaneously increasing antioxidant activity, which reduces water absorption from the rhizosphere (Liu et al., 2022). Regarding salinity stress tolerance, the selected strain VITK-3 demonstrated the ability to endure NaCl concentrations exceeding 20%, whereas VITK-1 exhibited tolerance up to 15% on MSM medium, indicating its comparatively lower resistance to high salinity. This salinity tolerance is crucial for effective PGPR colonization in the root zones, enabling nutrient assimilation and plant adaptation under abiotic stress conditions (Chu et al., 2019). The observed salt tolerance demonstrates the strain’s potential to endure different concentrations and intensities specific to alkaline soil conditions, since alkaline and saline conditions are commonly associated with high osmotic pressure, nutrient deficiencies, and disruption of metabolic processes. To investigate the presence of stress conditions, the isolated strains were tested across a range of tolerance levels. The selected strains exhibited optimal growth under alkaline conditions on MSM medium at pH levels ranging from 3 to 11 at 37 °C. These conditions represent alkaline soil stress, and the wide pH tolerance indicates the stability and adaptability of the strains to high pH soils under *in vitro* conditions. However, minimal growth was observed in *Pseudomonas* sp. and *Burkholderia* sp. related species when exposed to contaminated soils. Additionally, these strains were found to regulate micronutrient-transporting genes, which may contribute to their adaptability under extreme pH conditions (Hassen et al., 2018; Zhou et al., 2018; Dixit et al., 2020).

Finally, the selected strains exhibited resistance to heavy metals, highlighting their potential to thrive in contaminated soils and contribute to microbial community stability under environmental stress. However, the PGPR isolates showed a strong ability to solubilize and tolerate high concentrations of heavy metals, including cadmium, iron, copper, zinc, and lead. Under metal-affected alkaline soil conditions, these strains exhibit the ability to withstand heavy metals and stimulate their potential to maintain metabolic activity and support plant growth and development, which affects the availability and degradation of metals. This heavy metal tolerance further supports their role in promoting plant growth and maintaining soil health under abiotic stress conditions.

In current findings, the inoculation of PGPR significantly enhanced the growth and development of tomato seedlings under alkaline soil stress conditions. The results indicate that treatments with *Pseudomonas* sp. and *Burkholderia* sp. had a substantial positive impact on the plant fresh weight and dry weight of both roots and shoots (Hwang et al., 2021). Under alkaline conditions, comparable benefits were noted in several plant physiological and biochemical parameters. Notably, the photosynthesis rate significantly increased when seeds were treated with bacterial consortia as opposed to individual treatments and control groups (Kumar et al., 2022). This enhancement supports key physiological processes involved in chlorophyll pigmentation, electron transport, and gas exchange, thereby increasing carbon assimilation required for basic growth processes and contributing to improved stress tolerance under stress conditions (Ashraf and Harris, 2013). Additionally, this treatment helped alleviate osmotic stress induced by alkaline conditions as described in the previous report, without PGPR microbial treatments (Kumar et al., 2024). Similarly, research on *M. halliana* under salt-alkali stress conditions showed a reduction in metabolic pathways associated with protein expression (Zhu et al., 2020). Our findings suggest that consortium treatment maximised total protein content, thereby enhancing cell division under alkaline soil stress. In *Brassica rapa*, alkalinity stress has been associated with reduced plant biomass, increased oxidative stress, decreased photosynthetic activity, and nutrient absorption (Navarro-León et al., 2023). In bacterial inoculation studies, the synthesis of proline and bioactive compounds showed statistically significant differences (*p* < 0.05) under stress conditions. These plants exhibited enhanced enzymatic and non-enzymatic activity, particularly through the production of phenolics and flavonoids, which play a crucial role in abiotic stress tolerance, as previously reported (Burattini et al., 2017). The PGPR bacteria isolated in this study improved antioxidant enzymatic activity against salt stress by inhibiting reactive oxygen species (ROS). By upregulating crucial enzymatic activity, membrane permeability, and increasing the production of metallothionein, all of which support plant growth, regulate defense mechanisms against alkaline stress, maintain nutrient uptake, and sustain physiological stability in the presence of oxidative stress (Rajput et al., 2021; Iftikhar and Shah, 2025). Among the individual treatments and control groups, consortia treatments significantly enhanced antioxidant activity, along with other isolated strains such as *Pseudomonas* and *B. gladioli* species, ultimately supporting plant growth (Rahman et al., 2018; Khanna et al., 2019).

Our results revealed that nutrient analysis showed a slight but significant increase in nutrient uptake in tomato crops treated with bacterial consortia compared to individual strains and control groups under alkaline conditions. The availability of macronutrients, notably nitrogen and phosphorus, as well as essential elements iron, manganese, and zinc, increases nutrient accumulation during the early stage of seedling development. This increases nutrient availability in the rhizosphere’s soil and more efficient nutrient absorption by plant roots, reduces the negative impacts of alkaline stress, and strengthens beneficial plant-microbe interactions (Farghaly et al., 2022; Zhao and Zhang, 2025). While bacterial consortia treatments initially induced mild stress responses in plants, they ultimately contributed to enhanced tolerance against alkaline stress by influencing molecular regulatory mechanisms. Similar molecular-level responses and plant signalling pathways have been observed in *Puccinellia tenuiflora* and *Leymus chinensis* under stress conditions, highlighting the role of microbial interactions in stress adaptation (Guo et al., 2017).

Under alkaline stress, plants struggle to absorb essential nutrients, leading to restricted root growth and reduced water uptake from the rhizosphere. Our study indicates that bacterial treatments enhance the nutritional content of tomato seedlings under stress conditions, significantly affecting the expression of nutritional and stress response genes between treated and non-treated groups. Specifically, genes *NRT2*, *GR*, and *DRE* were upregulated in root-treated samples, while *PT1* and *AMT1* showed high regulation in treatments T2 and T3. These genes, involved in phosphate transport and nitrogen assimilation indicates enhanced nutrient uptake under alkaline stress conditions. This molecular response is consistent with the observed increases in nutrient uptake and root development in treated plants. *NRT2* is a key regulator of nitrate and ammonium transport, suggesting improved nitrogen metabolism and maintenance of water balance under stress. *GR* and *DRE* genes, which are associated with antioxidant enzyme activity (Mhamdi et al., 2010) through the production of superoxide dismutase and amylase, regulates stress-adaptive metabolic processes and correlates with enhanced chlorophyll pigmentation and root growth under stress conditions (Lata and Prasad, 2011). The upregulation of these genes in PGPR-treated plants enhances nutrient accumulation from the soil rhizosphere.

The research findings suggest that bacterial consortia have a more significant impact on plant growth and development compared to individual bacterial strains. This includes enhanced nutrient uptake, nitrogen fixation, and biofilm formation against phytopathogens, along with other bacterial activities. Overall, the bacterial consortia improved nutrient availability in the soil rhizosphere and stimulated phytohormone production, ultimately enhancing plant stress tolerance.

**Figure 7:**
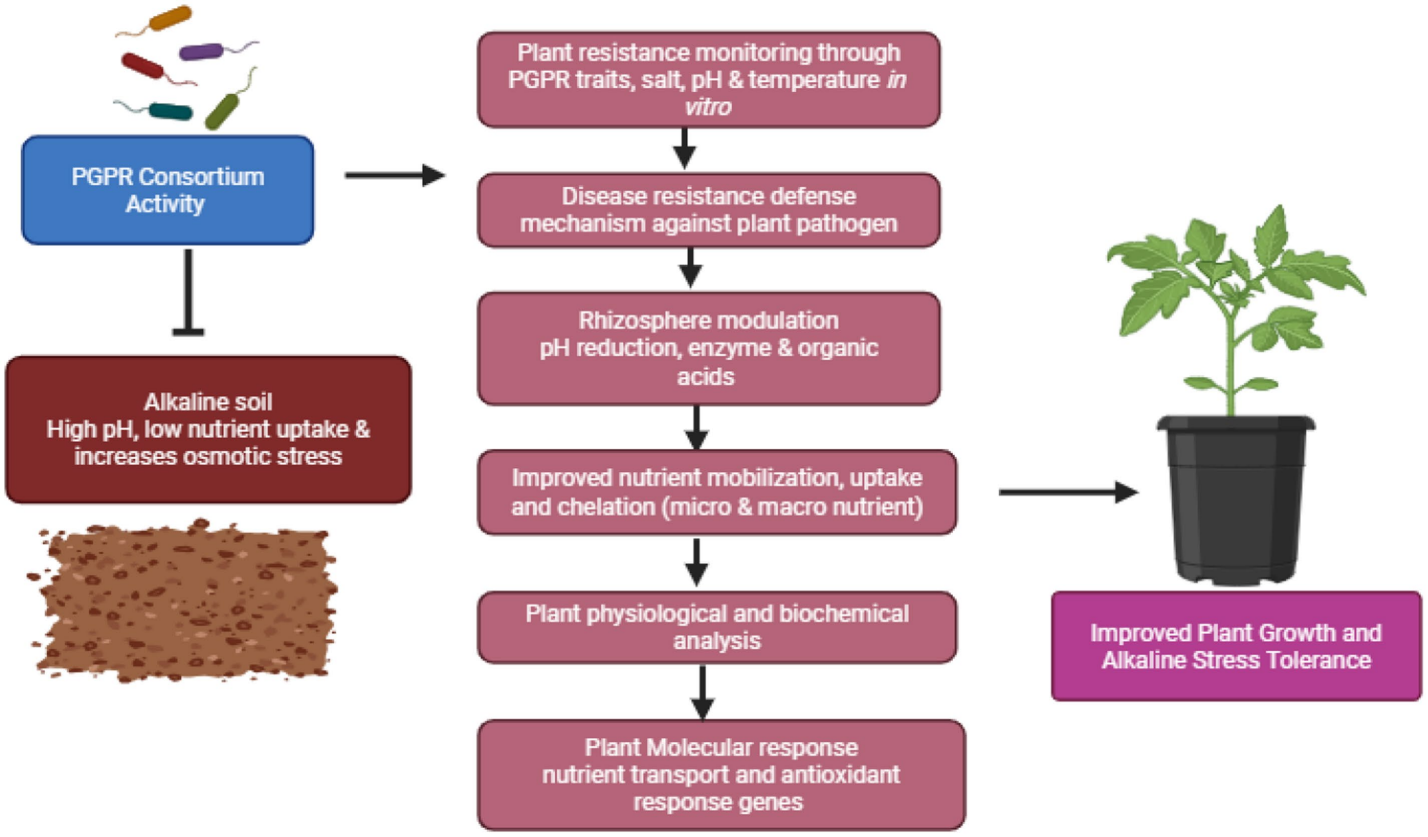
Schematic overview of the discussion process.

These results support the conceptual framework, which is schematically illustrated in Figure 7, demonstrating that bacterial consortia enhance tomato seedling tolerance to alkaline stress through modulation of rhizosphere function, nutrient uptake, and plant molecular responses. Under high pH conditions, the bacterial consortia improve nutrient availability by modulating rhizosphere processes, including phosphate solubilization, siderophore production, antagonistic activity, and the production of hydrolytic enzymes. These rhizosphere-mediated activities enhance plant defense mechanisms while simultaneously maintaining bacterial tolerance under metal-stress conditions. Consequently, the increased bioavailability of macro- and micronutrients promotes biomass accumulation, chlorophyll pigmentation, and enhanced antioxidant enzymatic activity, thereby reducing osmotic imbalance in treated plants under alkaline stress. These physiological changes are closely associated with molecular-level responses by the upregulation of genes involved in nutrient transport and stress adaptation. The activation of these stress-responsive genes enhances the efficiency of antioxidant enzymes, protecting plant cells from oxidative damage and maintaining membrane stability under stress conditions. Overall, these findings support the performance of consortium treatments compared to individual strains and control groups, highlighting their effectiveness in promoting plant growth and development under alkaline soil conditions.

Our study concludes that PGPR isolates from agricultural fields significantly enhance PGPR traits and extracellular activity, supporting bacterial survival under stress conditions. This enhancement improves nutrient availability in alkaline soil, offering an effective strategy for soil fertilization and agricultural productivity while addressing nutrient limitations from soil to plant roots. These advancements can contribute to large-scale crop production, ultimately benefiting human health. Although the microbial consortium substantially outperformed individual strains in controlled conditions, consortium-based systems may include trade-offs such as inter-strain competition, fluctuations in community stability over time, and unreliable performance in the field. To optimize nutrient acquisition from soil to plant roots, future research will focus on microbial consortia interactions at the gene expression level and metabolic pathways. The study will focus on evaluating tomato yield and fruit nutrient content, as well as assessing the impact of PGPR treatments on soil microbial communities, root metabolic regulation under diverse environmental conditions, and the broader suitability for agricultural systems.

## 5 Conclusion

This study successfully demonstrated isolated and characterized PGPR from agricultural soil. The results show that the two bacterial isolates, *Pseudomonas* sp. and *Burkholderia* sp., significantly improved seed germination, root and shoot length by enhancing both physiological and biochemical features in alkaline soil. These findings contribute to our understanding that these bacterial inoculants facilitate nutrient acquisition and alleviate plant growth limitations, proving effective as growth promoters for tomato seedlings. However, further research is needed to explore and optimize various microbial consortia formulations as a sustainable approach to enhancing crop yield and limiting nutrient mobilization in tomato cultivation across environmental soil conditions through long-term studies.

## Data Availability

The data presented in this study are publicly available. The data can be found at: https://www.ncbi.nlm.nih.gov/genbank, accession numbers OP102696 and PP897814.

## Glossary

PGPR: Plant growth promoting rhizobacteria
DDW: Double distilled water
CPG: Casamino Acid-Peptone-Glucose Broth
PDB: Potato dextrose broth
PDA: Potato dextrose agar
NBRIP: National Botanical Research Institute’s Phosphate
IAA: Indole-3-acetic acid
CAS: Chrome Azurol S
HCN: Hydrogen cyanide
NCBI: National Center for Biotechnology Information
BLAST: Basic local alignment search tool
MSM: Minimal salt medium
SDW: Sterile distilled water
PBS: Phosphate buffered saline
DPPH: 1,1, diphenyl-2-picrylhydrazyl
AlCl_3_: Aluminum chloride
qRT-PCR: Quantitative reverse transcription polymerase chain reaction
PCR: Polymerase chain reaction
DNA: Deoxyribonucleic acid
rDNA: Recombinant DNA
RNA: Ribonucleic acid
cDNA: Complementary DNA
TAQ: *Thermus aquaticus*
MgCl_2_: Magnesium chloride
DNTPs: Deoxynucleotide triphosphates
ANOVA: Analysis of variance
NAF: National Agro Foundation
SPAD: Soil plant analysis development
NaOCl: Sodium hypochlorite
Cd: Cadmium
Fe: Iron
Co: Cobalt
Zn: Zinc
Pb: Lead
NRT: Nitrate transporter
GR: Glutathione reductase
DREB: Dehydration responsive element binding
PT: Phosphate transporter
AMT: Ammonium transporter
NaCl: Sodium chloride
HCL: Hydrochloric acid
NaOH: Sodium hydroxide
Ofl: Ofloxacin
CIP: Ciprofloxacin
Gen: Gentamicin
Ceft: Ceftazidine
Tet: Tetracycline
Amox: Amoxicillin
Van: Vancomycin
Chl: Chloramphenicol
