# Supplementary material for: Bacterial consortia enhance nutrient uptakes and molecular response in tomato seedlings under alkaline soil stress: a comparative study
Source: Front Microbiol. 2026 Feb 26;17:1738650. doi: 10.3389/fmicb.2026.1738650 (PMC12979434; doi:10.3389/fmicb.2026.1738650)
Supplement: Supplementary file 1 [file Supplementary_file_1.pdf]

Supplementary Data

Supplementary Data 1. Seed germination equation of total germination rate, seedling vigor index, relative seed germination, and energy of germination.

$$\text{Total germination rate} = \frac{\text{number of germination seeds}}{\text{Total number of seeds tested}} \text{x100}$$

Seedling vigor index= Average shoot length + average root length x 100

$$\text{Relative seed germination} = \frac{\text{No. of germinated seed in treatment}}{\text{No. of germination seeds in control}} \text{X 100}$$

$$\text{Energy of germination} = \frac{\text{no. of germinated seeds after four days}}{\text{No. of germination days}}$$

Supplementary Data 2. Chlorophyll a and b

V = Volume of the plant extract with acetone (ml)

W = Weight of fresh tissue sample (g)

Equations:

Chlorophyll a (mg/g) = [12.7(OD663) – 2.69(OD650] x V/1000 x W

Chlorophyll b (mg/g) = [22.9(OD645) – 4.68(OD663) x V/1000 x W

mg total chlorophyll/g tissue = 20.2 (OD645) + 8.02 (OD663) x

V/1000 x W

Supplementary Data 3. Antioxidant activity of DPPH equation

DPPH percentage (%) inhibition = $[(A_B – A_A)/ A_B]$ x 100

A_B = Denotes the absorbance of DPPH radical + methanol (control)

A_A= Represents the absorbance of DPPH radical + sample extract

Supplementary Figure 1. Compatibility between the two bacterial isolates was assessed on nutrient agar using the agar-streak plate technique after 24 h of incubation. **(A)** *Pseudomonas sp.* VITK-1 (rod-shaped) streaked at the center of the plate with *Burkholderia sp.,* round it using a cotton swab. **(B)** *Burkholderia sp.,* VITK-3 (coccoid-shaped) streaked at the center with *Pseudomonas sp.,* spread around it, showing no inhibition zones, indicating compatibility. **(C)** Growth and interaction of both strains in nutrient broth after 24 h; cells were fixed with 2.5% glutaraldehyde and prepared for scanning electron microscopy (SEM) analysis. Plant growth-promoting traits were evaluated as follows: **(D)** Phosphate solubilization on NBRIP agar after 48 h of incubation, **(E)** Siderophore production on chrome azurol (CAS) agar using the agar diffusion method, indicated by yellow pigmentation **(F)** Ammonia production detected using Nessler's reagent with freshly grown cultures, **(G)** Hydrogen cyanide (HCN) production on glycine-supplemented nutrient agar after four days of incubation, indicated by orange pigmentation, and **(H-I)** Nitrogen fixation ability assessed on Jensen's agar medium after 48 h of incubation.

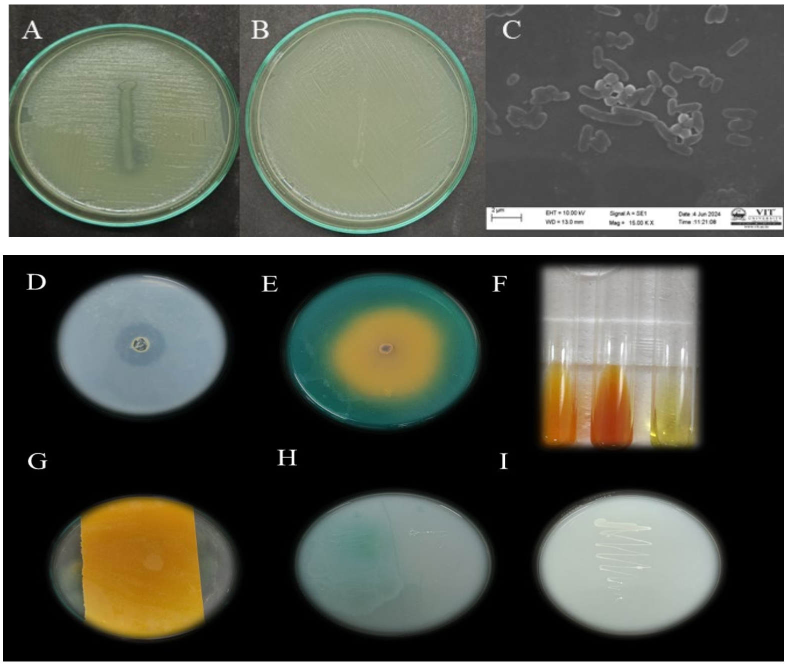

Supplementary Figure 2. *In vitro* indole-3-acetic acid (IAA) production by two plant growth-promoting rhizobacteria (PGPR) isolates (VITK-1 and VITK-3) cultured in nutrient broth supplemented with tryptophan. IAA concentration was quantified after incubation and compared with an uninoculated control. Bar graphs represent the mean and standard error (SE). Different letters show statistically significant differences among treatments as determined by one-way ANOVA, $p<0.05$.

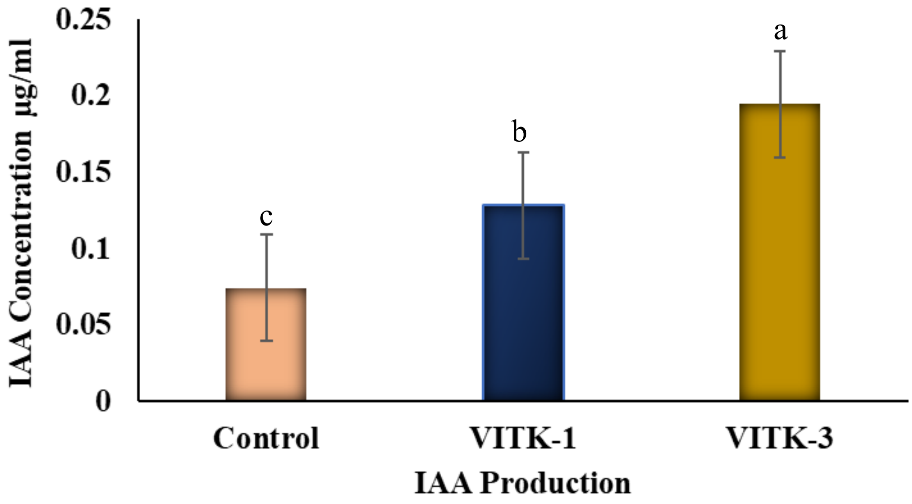

Supplementary Figure 3. Effect of plant growth-promoting rhizobacteria (PGPR) on in vitro growth and germination of tomato seedlings. **(A)** Seed priming with bacterial suspension on the number of germinated seeds over 12 days, compared with the untreated control. **(B)** Germination percentage of tomato seeds following PGPR treatment. Control seeds were treated with double-distilled water (DDW). Data represented as mean ± SD. Different letters indicate statistically significant differences among treatments, according to the Student's t-test and Duncan's multiple tests, with significance levels of $p<0.05$ and $p<0.0001$.

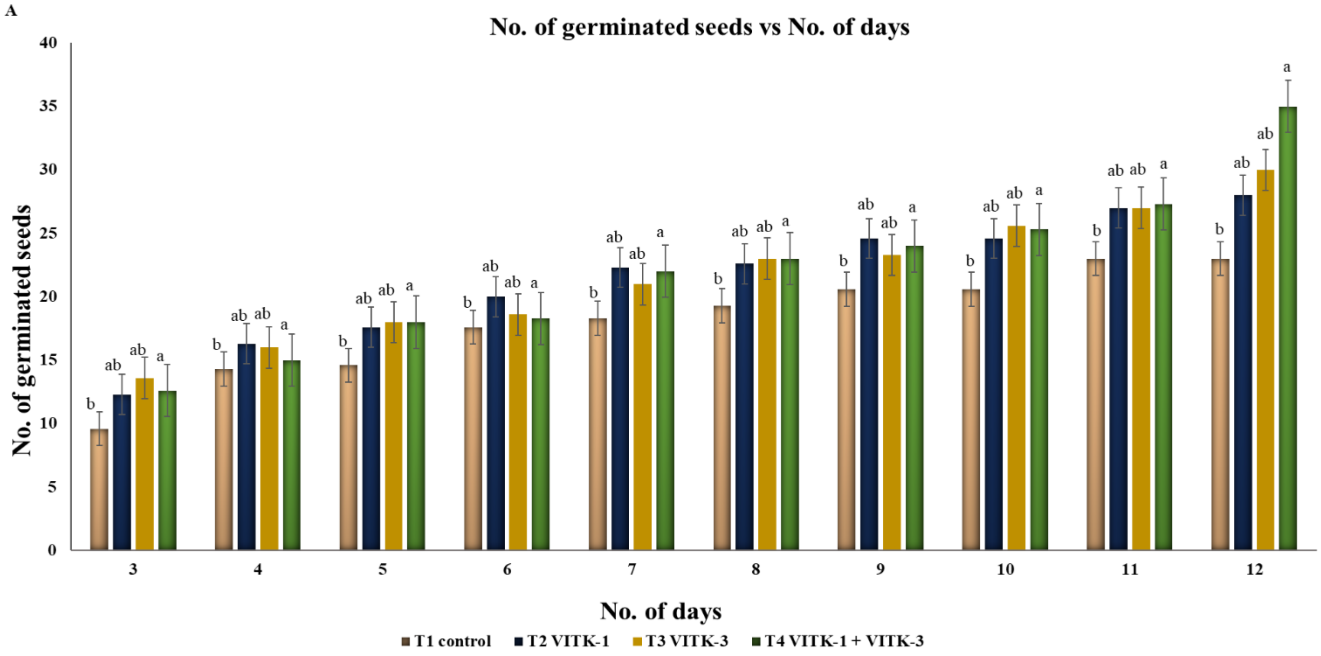

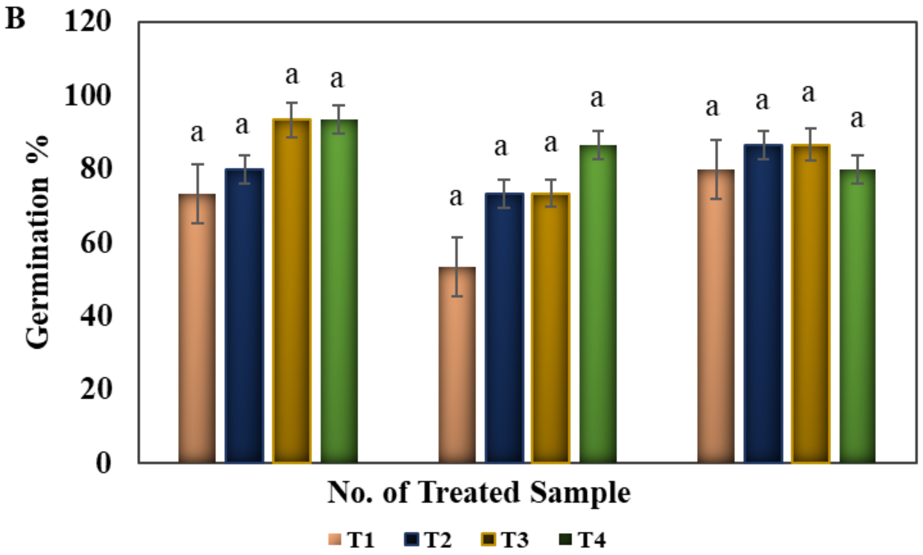

Supplementary Figure 4. Effect of individual and combined PGPR strains on tomato seedlings' germination and early growth analysis. Tomato seeds were treated with bacterial suspensions of *Pseudomonas sp.* (VITK-1, T2), *Burkholderia sp.* (VITK-3, T3), and a consortium (VITK-1 + VITK-3, T4) by spraying, and the control (T1) seedling served as sterile distilled water. Seeds were germinated on sterile petri plates and monitored for 10-12 days. The representative seedlings from each treatment are shown as **(A)** control, **(B)** VITK-1, **(C)** VITK-3, and **(D)** VITK-1 + VITK-3 consortium. Seedlings treated with bacterial suspensions exhibited enhanced root and shoot growth compared with control and individual strain treatments.

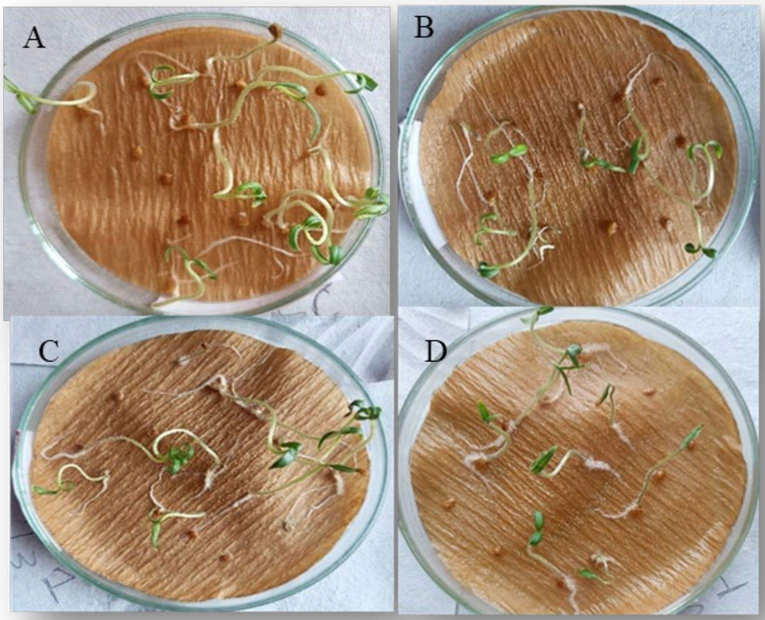

Supplementary Figure 5. Experimental design for bacterial suspension study under greenhouse conditions. Tomato seeds were initially treated by immersion in a freshly prepared bacterial suspension and sown in pro trays for germination. The experimental treatment consisted of: T1 (control with DDW), T2 (VITK-1), T3 (VITK-3), and T4 (consortium VITK-1 + VITK-3). Seedlings were subsequently transplanted into pots containing 5-7 kg of soil with 3 biological replicates, which were analyzed for pH before planting. Plants received 10 ml of freshly prepared bacterial suspension once per week throughout the experimental period, while control plants were maintained without bacterial treatment. Greenhouse conditions were maintained at controlled temperature and humidity. At the end of the experiment, plant samples were collected for analysis of macro-and micronutrient content, physiological parameters, biochemical characteristics, and expression of nutrient-regulating and stress-response genes.

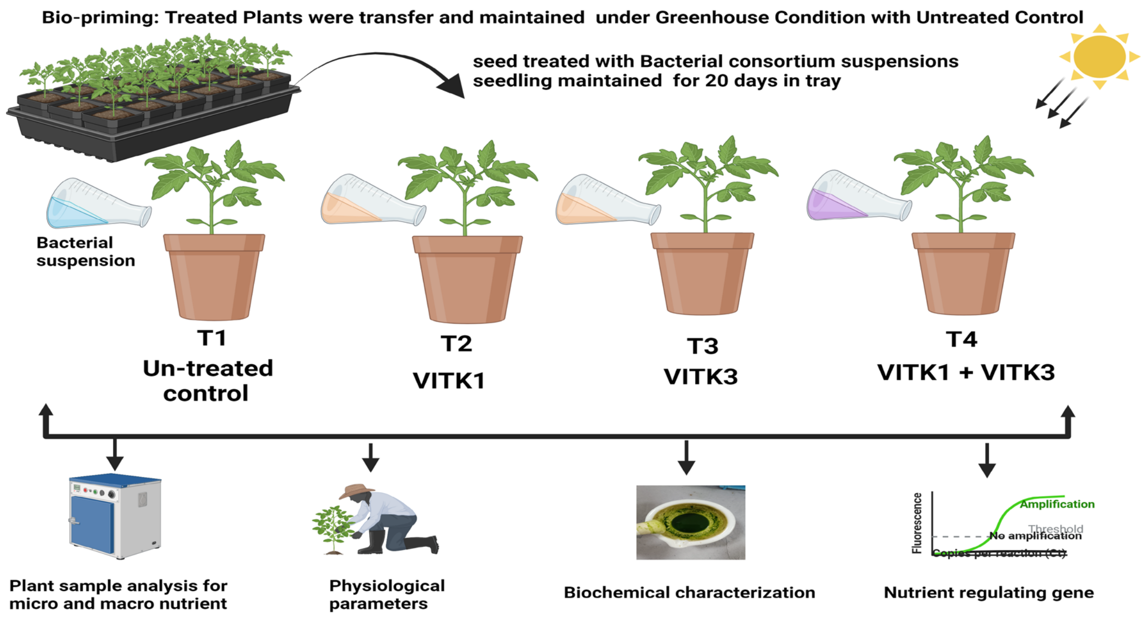

Supplementary Figure 6. Root growth performance of tomato seedlings under bacterial suspension treatments. **(A)** Control seedlings treated with sterile distilled water. **(B)** Seedlings inoculated with isolate VITK-1. **(C)** Seedlings inoculated with isolate VITK-3. **(D)** Seedlings treated with a consortium of VITK-1 and VITK-3. The image illustrated the effect of individual and combined bacterial inoculants on root system development, length and root formation was compared with the control group under alkaline soil conditions. This is associated with improvement in plant physiological parameters.

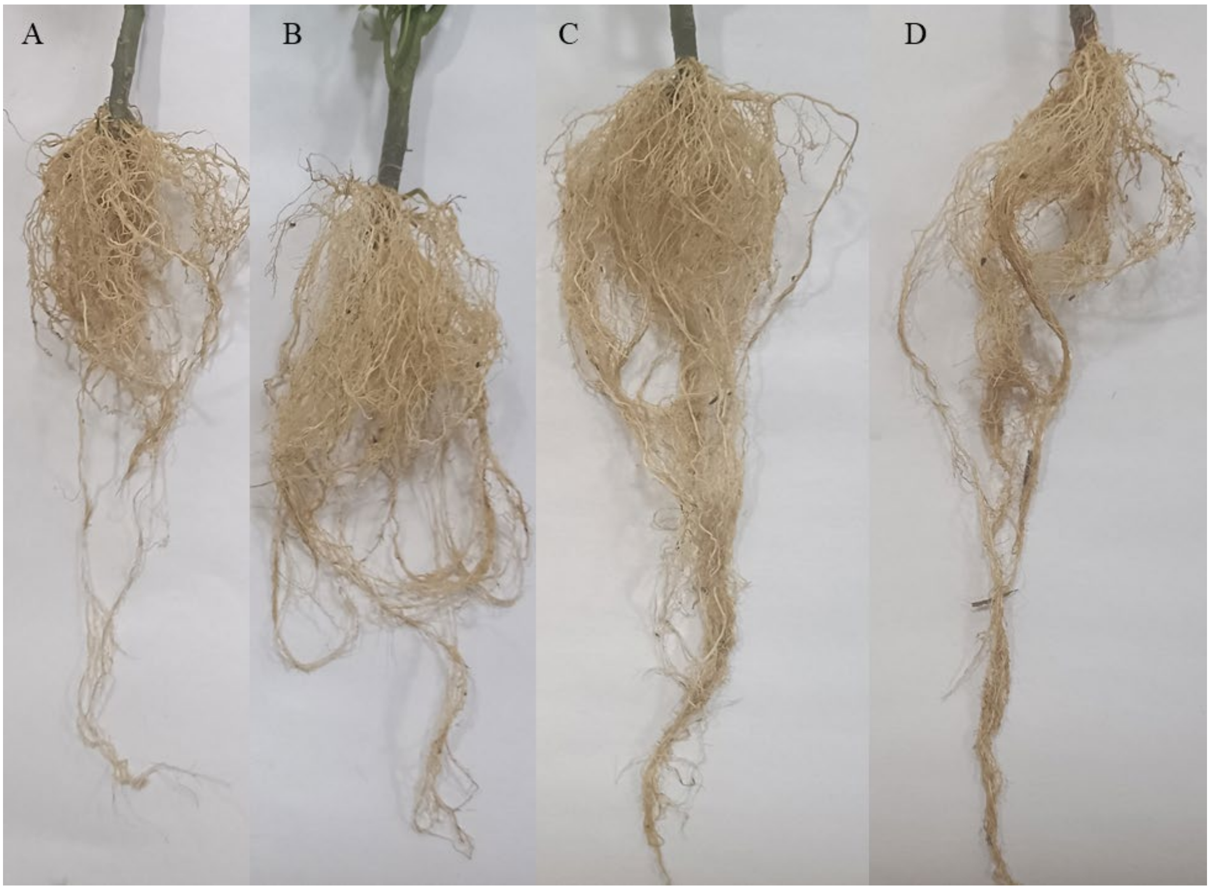

Supplementary Table 1. Primer sequence utilized for qRT-PCR for gene expression analysis

Gene name	Primer sequence (5'-3')	Product size	Gene function
PT1	CAGCATTCAAGGGCGCATTC CAGCAGGGATTGCACCAAAC	117bp	Represents the phosphate solubilization and efficiency
GR	TCCCATCGGCTCTGAAGTTAGTGGG TCTTTGCATCCTCCAGTTCTGGCCC	119bp	Indicates a stress-related transcript gene
AMT1	GTTGTGGTGCATGGGGGATA TATGCGCCCCGAGTAGTTTC	135bp	Transport of nitrogen and ammonia
NRT2	CCCGTTCACTGCTTGGAGAA GAAGCGAGGTTACCATCAGGT	111bp	Transport of Nitrate
DRE	GGAGAGCAAAACAGCAGAAG CAAAAATCCACTGGCCTAGC	467bp	Indicates the drought tolerance transport gene
ACTIN	TAAAAGTGCGAGTGTCCTGT CAGGCACCTCTCAAGTATGT	150bp	Housekeeping gene

Supplementary Table 2. Qualitative characterization of plant growth-promoting rhizobacteria (PGRP) using plate assay techniques. Results represent the semi-scoring system, where "-

“indicates a negative result (no detectable activity), “+++” indicates a moderately positive result, and “++++” indicates a highly positive result. Phosphate solubilization was assessed based on the formation of a clear zone around the bacterial colony. Ammonia production was determined by color development in the assay medium. Siderophore and hydrogen cyanide (HCN) production were indicated by the color pigmentation. Nitrogen fixation ability was evaluated by bacterial growth on nitrogen-free medium. Extracellular enzyme activities, including protease, cellulase, amylase, lipase, and gelatinase, were determined based on zone formation and color pigmentation in the respective assay media.

S. No	Qualitative Screening of PGPR	*Pseudomonas sp.* (VITK-1)	*Burkholderia sp.* (VITK-3)
1	Phosphate Solubilization	++++	++
2	Ammonia Production	++++	++++
3	Siderophore Production	++++	+++
4	Hydrogen Cyanide	++++	-
5	Nitrogen Fixation	++++	+++
6	Protease	++++	+++
7	Cellulase	++++	+++
8	Amylase	++++	+++
9	Lipase	++++	++++
10	Glycine	++++	+++

Supplementary Table 3. *In vitro* screening of two bacterial (VITK-1 and VITK-3) strains for compatibility, antagonistic activity, and stress tolerance. The results are represented using a semi-scoring system. Compatibility between the two bacterial strains was assessed, where “++++” indicates no antagonistic activity (full compatibility). Antagonistic interaction against phytopathogens was determined as a percentage inhibition, where *Fus* refers to *Fusarium oxysporum* and *Ral* refers to *Ralstonia solanacearum*. The stress tolerance of the bacterial strains was evaluated based on their ability to grow in the presence of 20% NaCl, at a temperature of 37°C, and at a pH of 9. Hemolytic activity was assessed on blood agar medium, where “- “indicates the absence of hemolysis, confirming the non-pathogenic nature.

Strains	Compatibility Activity	Antagonistic Activity	NaCl salt Tolerance	Temperature	pH	Hemolytic Activity
Pseudomonas sp. VITK-1	++++	76.5% **Fus*<br>71.8% **Ral*	20%	37 °C	pH: 9	_
Burkholderia sp. VITK-3	++++	64.1% **Fus*<br>35.7% **Ral*	20%	37 °C	pH: 9	_

Supplementary Table 4. *In vitro* screening of two bacterial strains (VITK-1 and VITK-3) for heavy-metal tolerance at different concentrations (ppm) of cadmium (Cd), copper (Cu), Zinc (Zn), lead (Pb), and iron (Fe). Bacterial tolerance was evaluated based on the growth response in the presence of each metal. Results are expressed using a semi-scoring system, where "+++" indicates strong bacterial growth, "++" indicates moderate growth, and "-"indicates no detectable growth of the isolates.

Strains	Cadmium (ppm)			Copper (ppm)			Zinc (ppm)			Lead (ppm)			Iron (ppm)		
	250	500	1000	250	500	1000	250	500	1000	250	500	1000	250	500	1000
Pseudomonas sp. VITK-1	+++	+++	+++	+++	+++	+++	+++	+++	-	+++	+++	+++	+++	+++	+++
Burkholderia sp. VITK-3	+++	+++	+++	+++	+++	+++	+++	+++	-	+++	+++	+++	+++	+++	+++
